# An Overview of the Protein Binding of Cephalosporins in Human Body Fluids: A Systematic Review

**DOI:** 10.3389/fphar.2022.900551

**Published:** 2022-06-28

**Authors:** C. Jongmans, A. E. Muller, P. Van Den Broek, B. De Melo Cruz De Almeida, C. Van Den Berg, J. Van Oldenrijk, P. K. Bos, B. C. P. Koch

**Affiliations:** ^1^ Department of Hospital Pharmacy, Erasmus University Medical Center, Rotterdam, Netherlands; ^2^ Department of Medical Microbiology and Infectious Diseases, Erasmus University Medical Center, Rotterdam, Netherlands; ^3^ Department of Medical Microbiology, Haaglanden Medical Center, The Hague, Netherlands; ^4^ Department of Orthopedics and Sports Medicine, Erasmus University Medical Center, Rotterdam, Netherlands

**Keywords:** cephalosporins, protein binding, human, body fluids, pharmacokinetics, systematic review

## Abstract

**Introduction:** Protein binding can diminish the pharmacological effect of beta-lactam antibiotics. Only the free fraction has an antibacterial effect. The aim of this systematic literature review was to give an overview of the current knowledge of protein binding of cephalosporins in human body fluids as well as to describe patient characteristics influencing the level of protein binding.

**Method:** A systematic literature search was performed in Embase, Medline ALL, Web of Science Core Collection and the Cochrane Central Register of Controlled Trials with the following search terms: “protein binding,” “beta-lactam antibiotic,” and “body fluid.” Only studies were included where protein binding was measured in humans *in vivo*.

**Results:** The majority of studies reporting protein binding were performed in serum or plasma. Other fluids included pericardial fluid, blister fluid, bronchial secretion, pleural exudate, wound exudate, cerebrospinal fluid, dialysate, and peritoneal fluid. Protein binding differs between diverse cephalosporins and between different patient categories. For cefazolin, ceftriaxone, cefpiramide, and cefonicid a non-linear pattern in protein binding in serum or plasma was described. Several patient characteristics were associated with low serum albumin concentrations and were found to have lower protein binding compared to healthy volunteers. This was for critically ill patients, dialysis patients, and patients undergoing cardiopulmonary bypass during surgery. While mean/median percentages of protein binding are lower in these patient groups, individual values may vary considerably. Age is not likely to influence protein binding by itself, however limited data suggest that lower protein binding in newborns. Obesity was not correlated with altered protein binding.

**Discussion/Conclusion:** Conclusions on protein binding in other body fluids than blood cannot be drawn due to the scarcity of data. In serum and plasma, there is a large variability in protein binding per cephalosporin and between different categories of patients. Several characteristics were identified which lead to a lower protein binding. The finding that some of the cephalosporins display a non-linear pattern of protein binding makes it even more difficult to predict the unbound concentrations in individual patients. Taken all these factors, it is recommended to measure unbound concentrations to optimize antibiotic exposure in individual patients.

**Systematic Review Registration:** PROSPERO, identifier (CRD42021252776).

## 1 Introduction

Cephalosporins are a frequently used class of beta-lactam antibiotics in hospitalised patients. They are used for a broad spectrum of indications and in many types of patients, from peri-operative prophylactic use in relatively healthy patients to therapeutic use in critically ill septic patients. The efficacy of the cephalosporins is best correlated to the “time that the free (*f*) concentration is above the minimum inhibitory concentration (MIC) of the pathogen (*f*T > MIC).” The *f*T > MIC is one of the pharmacokinetic (PK)/pharmacodynamic (PD)-indices to describe antimicrobial effect. Importantly, all these indices are based on free concentrations, i.e., the fraction of the total concentration that is not bound to proteins. This underlines the importance of protein binding for antimicrobial efficacy.

Protein binding can decrease the pharmacological effect of beta-lactam antibiotics, since only the unbound fraction is pharmacologically active (Craig and Kunin, 1976; Merrikin et al., 1983; Beer et al., 2009; Briscoe et al., 2012). The binding to proteins depends on the number of binding sites as well as binding affinity in relation to the antibiotic concentration. Besides its impact on the antibacterial effect, protein binding also affects the pharmacokinetics, since it has an influence on drug clearance and distribution (Roberts et al., 2013).

It is essential that antibiotics distribute throughout the body since most infections do not occur in the bloodstream. However, only the unbound fraction is able to penetrate into the extravascular space (Tan and Salstrom, 1977). From the extravascular space, the drug will distribute into tissues or be metabolised or excreted from the body (Wise, 1983). The unbound blood concentration will equilibrate with unbound concentration in the extravascular space (Bergogne-Bérézin, 2002). Therefore, it is hypothesised that unbound concentrations reflect the active concentrations at the site of infection.

The level of protein binding is often regarded as a fixed percentage per antibiotic, i.e., independent of the patient disease state and antibiotic concentrations. With increasing knowledge, we nowadays know that protein binding is often more complex. Some antibiotics/drugs display a concentration-dependent protein binding. As a result, proteins are being saturated in high concentrations of antibiotics, resulting in an increased unbound fraction. However, the opposite effect was also shown with lower unbound fractions at high antibiotic concentrations (Bulik et al., 2010). In specific clinical conditions, protein binding might change, such as in critically ill patients or in dialysis patients (Steele et al., 1979; Wilkes et al., 2019; Jager et al., 2020).

Since protein binding is crucial for antibacterial efficacy as well as distribution to the site of infection, the aim of our systematic literature review was to give an overview of the current knowledge on protein binding of cephalosporins in human body fluids as well as to describe characteristics influencing the level of protein binding based on clinical studies in humans.

## 2 Materials and Methods

We followed the PRISMA Statement as a guideline. This systematic review is registered under PROSPERO CRD42021252776.

### 2.1 Search Strategy and Data Sources

The final database consisted of articles, which were included in three stages. First, on 12 April 2021, studies were identified by searching four databases (Embase, Medline ALL, Web of Science Core Collection and Cochrane Central Register of Controlled Trials) with no restriction on publication date. Duplicates were removed. The search strategy was developed by a biomedical information specialist from the medical library at the Erasmus University Medical Centre (Supplementary Appendix S1). The following search terms were used for the search strategy: protein binding and beta-lactam antibiotic and body fluid. The second stage consisted of an additional search (December 2021) to check the results of the first search for completeness. This search was performed in Pubmed using the name of the specific cephalosporin and unbound concentration as search terms. The third stage was performed during data extraction and consisted of checking the reference lists of the selected articles for missing articles.

### 2.2 Eligibility Criteria and Study Selection

Studies were included if they reported protein binding of cephalosporins in human body fluids. Included were human studies on cephalosporins in blood or other body fluids, measuring and reporting protein binding or measuring both total and unbound concentrations in actual study settings. Reviews, *in vitro* studies, animal studies, studies in tissues, conference papers and abstracts, and articles not in English were excluded. The primary outcome measure was the degree of protein binding of a beta-lactam antibiotic in a body fluid. In this review, we focus on cephalosporins, one class of beta-lactam antibiotic, as information on protein-binding of these antibiotics was most important for clinical care.

In the first stage, two reviewers screened the references independently according to the method of Bramer et al. (2017). The articles were included based on title and abstract (CJ and PB). In case of uncertainty based on title and abstract, the articles were included for full-text screening. Subsequently, full-texts were screened (CJ and BD). The reasons for exclusion after screening the full-text were noted. For the second and third stages, one reviewer screened the abstracts and full-texts (AM). A second reviewer screened the selected full-texts (CJ). In case of a disagreement between the reviewers, consensus was reached through discussion or if necessary, a third reviewer was consulted to make the final decision (BK).

### 2.3 Data Extraction Process

One reviewer (CJ) extracted data from the included articles. The following data were collected from the included studies in a data extraction form: 1) which antibiotic was used, 2) body fluid in which protein binding was measured, 3) population in which protein binding was measured, 4) number of participants, 5) dosage, 6) time point in which protein binding was measured, 7) sampling method, 8) analytical method, 9) percentage of protein binding, and (10) study type. A second reviewer (AM) checked the data extracted by the first reviewer. In case of a disagreement between the reviewers, consensus was reached through discussion or if necessary, a third reviewer was consulted to make the final decision (BK). With the final dataset the article selection was checked for duplicate publications.

The degree of protein binding was noted as a percentage. If an article noted the unbound fraction, this was converted to percentage of protein binding using the following equation: protein binding=1−unbound fraction*100% . When only unbound and total concentrations were known protein binding was also calculated $\left(protein\;binding=1-\frac{unbound\;concentration}{total\;concentration}\text{∗}100\text{\%}\right)$ .

## 3 Results

### 3.1 Background Information on Protein Binding and Its Relevance

**FIGURE 1:**
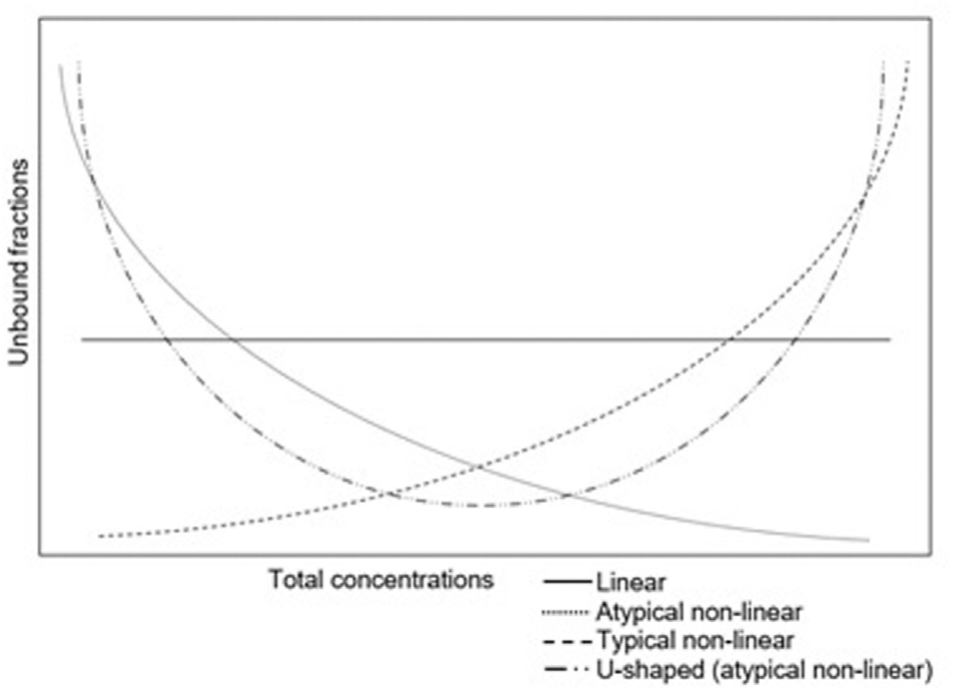
Different patterns of protein binding.

Various patterns of protein binding have been described. The degree of protein binding is often reported as a single value. In this situation the unbound fraction remains the same, irrespective of the total concentration. This so called “linear protein binding” is caused by large binding capacities of proteins or low binding affinities of drugs for proteins. At high total concentrations, proteins are still not saturated (Deitchman et al., 2018). However, in clinical practice with an increasing concentration, proteins might become saturated. This results in concentrations dependency and a non-linear protein binding. The most common pattern of non-linear protein binding has an increased unbound fraction with high total concentrations due to saturated binding sites on the proteins (typical non-linear protein binding). However, also the opposite pattern has been described: a high unbound fraction at low concentrations (atypical non-linear protein binding) (Muralidharan et al., 2005; Zhou et al., 2017). This has been described for the tetracyclines. An even more complex pattern has been described for tigecycline, which is U-shaped (Mukker et al., 2014; Dorn et al., 2018). This atypical pattern was linked to divalent metal ion chelation (Singh et al., 2016). The different patterns of protein binding are shown in Figure 1.

#### 3.1.1 Factors Affecting Level of Protein Binding

The most important protein for protein binding of antibiotics is albumin. Therefore, a decreased level of albumin is thought to significantly reduce the level of protein binding. Several clinical characteristics are known to influence the level of albumin. Low levels can be caused by specific situations such as systemic inflammatory response syndromes, hepatic diseases, aging, malnutrition, malignancy, burns, pregnancy, diabetes mellitus, infection, or pulmonary oedema. Otherwise, the albumin level can be within the normal range, but binding is altered due to displacement from protein binding sites by for example, bilirubin, urea or co-administered drugs (Tillement et al., 1978; Roberts et al., 2013).

An effect of temperature as well as pH on protein binding has been described previously. However, the physiological variation of both temperature and pH are limited (Hinderling and Hartmann, 2005). Therefore, the influence of both factors might be relevant in *in vitro* experiments, but not in a clinical setting (Kochansky et al., 2008).

Interaction with co-administered drugs might also influence protein binding. If two highly protein bound drugs are administered simultaneously, competition for the binding capacity will occur since there is a limited amount of protein available. This will lead to a higher free fraction of the drugs compared to the situation after administration of the drugs alone.

#### 3.1.2 Effects of Changes in Protein Binding on the Pharmacokinetics

Especially in hydrophilic drugs, an increased unbound concentration might result in an increased clearance. In addition, a change in protein binding is a major factor influencing the volume of distribution because only the unbound fraction is able to penetrate into the extravascular space (Tan and Salstrom, 1977; Nix et al., 1991). The change in volume of distribution is linearly correlated with the unbound fraction (Nix et al., 1991).

#### 3.1.3 Clinical Relevant Changes in Protein Binding

The changes in pharmacokinetics, as described, might result in an altered half-life, (unbound) concentrations in steady state, penetration into tissues and bioavailability (Schmidt et al., 2010). The degree and pattern of protein binding is likely to change the unbound concentrations over time during a dosing interval. These unbound concentrations are the parameters of interest to determine the clinical efficacy as well as potential toxicity. Drugs with an increased unbound concentration might be more effective at the beginning of the dosing interval. However, as a result of the increased clearance with the increased unbound concentrations, the antibiotic might be cleared faster with consequently inadequate concentrations at the end of the dosing interval. The magnitude of these changes might differ per antibiotic and in special populations. Some changes might lead to the need for altered drug dosing.

Overall, changes in protein binding are assumed to be of relevance mainly in highly protein bound drugs, since changes for those drugs will have a larger effect. Highly protein bound is defined as 90%–99.9% protein binding (Wright et al., 1996). A non-linear pattern of protein binding complicates the interpretation, since the unbound concentration-time profile will be less predictable (Deitchman et al., 2018). Notably, to predict the efficacy of an antibiotic dosing regimen, the unbound concentration-time profile needs to be correlated with the minimum target concentration required for antibacterial effect.

### 3.2 Systematic Review on the Protein Binding of Cephalosporins

#### 3.2.1 Study Selection

**FIGURE 2:**
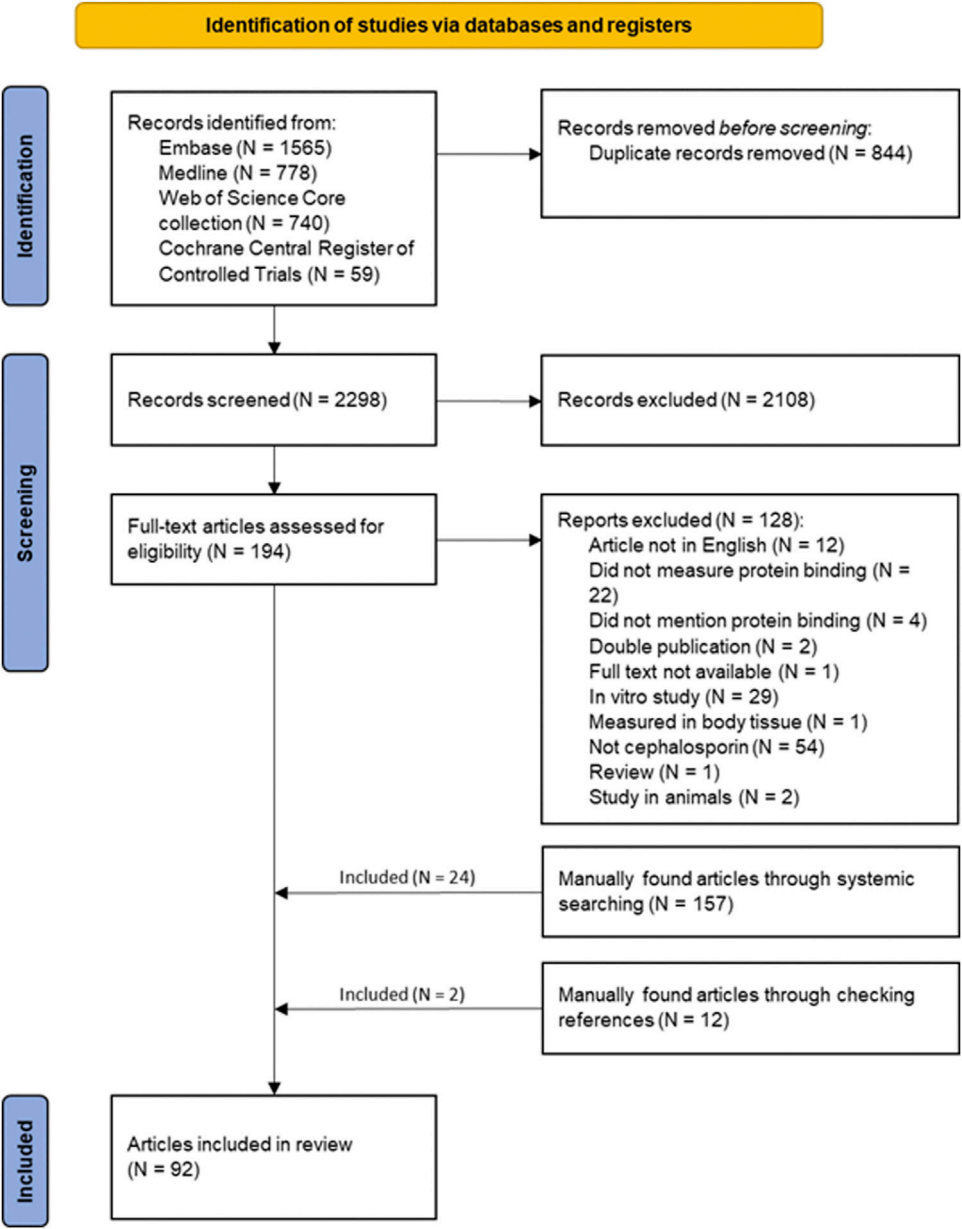
Flowchart of the identification of studies *via* databases and registers (N = number of articles).

In the first stage a total of 3,142 studies were identified. After duplicate removal (N = 844), 2,298 studies were screened on title and abstract, resulting in 194 primary studies. The full-text was not available for one of them. Of the remaining studies, 128 were excluded for the following reasons: not in English, double publication, did not measure protein binding, did measure protein binding but did not mention it, measured protein binding in body tissue, in animals or *in vitro*, review and not cephalosporins (Figure 2). The remaining 66 studies were included in the systematic review. During the second stage, 157 studies were screened of which 24 were eligible for inclusion. From the third stage, two studies were included out of 12 screened articles. This resulted in a total of 92 eligible for inclusion for this systematic review.

#### 3.2.2 Study Results

Table 1 gives an overview of the studies in which protein binding of cephalosporins was determined. The compromised version of the data extraction is available in this article whereas the full version of the data extraction is available in Supplementary Appendix S2.

**TABLE 1:** The extracted data of the included studies (detailed information in supplement).

Drug	References	Population (number of participants)	Analytical method	Protein binding (mean ± SD; or as indicated)	Pattern of protein binding (as reported, in the range in the studied-concentrations)
First generation cephalosporins					
Cefazolin in pericardial fluid	Nightingale et al. (1980)	Patients undergoing coronary artery bypass or cardiac valve replacement (16)	UF and microbiological assay	83.8% ± 4.1%	
Cefazolin in wound exudate	Rowan et al. (2017)	Burn and trauma patients with negative pressure wound therapy (8)	UF and HPLC-UV	44.6% ± 23.2%	
Cefazolin in plasma	Allegaert et al. (2009)	Pregnant women (30)	UF and HPLC-UV	M:75% (R:59–86%)	Non-linear
	Asada et al. (2018)	Patients undergoing cardiothoracic surgery with CPB (27)	UF and HPLC-UV	Preoperative: M:79%	
	Booke et al. (2021)	Critically ill patients on ECMO (6)	UF and HPLC-UV	During CPB: M: 55% 51% ± 9%	
	Deguchi et al. (1988)	Newborn infants at NICU (20)	UF and HPLC	49% ± 0.17	
	Dhanani et al. (2019)	25 year old female on ECMO (1)	UF and HPLC-MS/MS	After 1 h: 52%	Non-linear
				After 4 h: 60%	
				After 8 h: 60%	
	Dorn et al. (2019)	Morbidly obese (15) and non-obese patients (15)	UF and HPLC-UV	M:69.1% at high total concentration (132 mg/L) and M:78.4% at low total concentrations (14.4 mg/L)	
	Douglas et al. (2011)	Patients (semi)elective abdominal aortic aneurysm open repair surgery (12)	UF and LC-MS/MS	M:87% (I:74–90%)	
	Lantinga et al. (2016)	Patients scheduled to undergo percutaneous aspiration sclerotherapy of a symptomatic non-infected, non-neoplastic hepatic cyst (8)	UF and HPLC-UV	M:74.2% (I:71.8–81.2%)	
				At peak: M:69.2%	
				During cyst drainage: M:73.6%	
				Last sample: M:77.4%	
	Naik et al. (2017)	Patients undergoing urological or multilevel spine surgery (20)	Method for separation bound/free not known and UHPLC-MS/MS	69% (R:44–80%)	
	Palma et al. (2018)	Morbidly obese patients (4)	UF and HPLC-UV	M:68.3%	
	Parker et al. (2016)	Patients with peritoneal dialysis-associated peritonitis (1)	UF and UHPLC-MS/MS	R:79–82%	
	Roberts et al. (2015)	Critically ill adult patients (30)	UF and HPLC-MS	60% ± 12% at 5 min	
				73% ± 10% at 1 h	
				77% ± 9% at 6 h	
	Roberts et al. (2016)	Patients with peritoneal dialysis-associated peritonitis (11)	UF and UHPLC-MS/MS	M:63.9% (I:57.6–66.3%)	
	Rowan et al. (2017)	Burn and trauma patients with negative pressure wound therapy (8)	UF and HPLC-UV	86.4% ± 6.9%	
	Van Kralingen et al. (2011)	Morbidly obese patients scheduled to undergo laparoscopic gastric banding or gastric bypass surgery (20)	UF and HPLC-UV	M:79% (I:74–82%)	Non-linear
	Vella-Brincat et al. (2007)	Patients treated with cefazolin iv by continuous infusion or intermittent injection (31)	UF and HPLC-UV	Study I: 81.4% (R:49.4–90.2%)	Non-linear
				Study ii: trough 81.3% and peak 68.7%	
				From low concentrations (8.5 mg L^−1^): 91%–49% at high concentrations (140 mg L^−1^)	
Cefazolin in serum	Brodwall et al. (1977)	Patients with impaired renal function (9)	ED and microbiological assay	86.9% ± 1.84	
	Craig et al. (1973)	Healthy volunteers (6) and patients with various degrees of renal impairment (11)	ED and microbiological assay	Healthy volunteers: 83.9% ± 1.4%	
				Patients: R:61.6–84.7%	
	de Cock et al. (2017)	Children undergoing cardiac surgery with CPB (56)	UF and HPLC-UV	M:72% (I:64–77%)	Non-linear
	Himebauch et al. (2014)	Children undergoing CPB (12)	UF and HPLC-MS	M: 84.8% (I: 79.8–88.0%)	
				Lower during DHCA: M:78.9% (I:77.3%–81.9%)	
	Hites et al. (2016)	Patients undergoing gastric bypass surgery, partial hepatectomy, duodenopancreatectomy or colectomy (63)	UF and UPLC-UV	BMI < 35: 78.4%	
				BMI ≥ 35: 79.1%	
				TBW < 120 kg: 79.8%	
				TBW ≥120 kg: 79.6%	
	Hollis et al. (2015)	Patients with CPB (10)	UF and HPLC	R:53–75%	Linear
	Koshida et al. (1987)	Children, age 3–12 years undergoing examination with cardiac catheterization (5)	UF and HPLC-UV	78.1% ± 2.5%	
	Ohashi et al. (1986)	Patients with cirrhosis (12) or hepatitis (8) and normal volunteers (12)	UF and microbiological assay	Cirrhotic patients 72.3 ± 8.5%	
				Hepatitis patients 84.7 ± 3.3%	
				Control group 88.6 ± 1.9%	
Cefradine dialysate	Martea et al. (1987)	54 years male with continuous ambulatory peritoneal dialysis for end-stage renal failure with peritonitis (1)	UF and HPLC	6.1% ± 2.8%	
Cefradine pericardial fluid	Nightingale et al. (1980)	Patients undergoing coronary artery bypass or cardiac valve replacement (17)	UF and microbiological assay	<10%	
Cefradine plasma	Martea et al. (1987)	54 years male with continuous ambulatory peritoneal dialysis for end-stage renal failure with peritonitis (1)	UF and HPLC	29.1% ± 6.6%	
Cefalothin in pericardial fluid	Green et al. (1981)	Pediatric patients (2–12 years) (32)	UF and microbiological assay	79.6% ± 5.7% (<1 h after the administration in pericard eff.)	
Cefalothin in plasma	Parker et al. (2016)	Patients with peritoneal dialysis-associated peritonitis (1)	UF and UHPLC-MS/MS	R:47–51%	
	Roberts et al. (2016)	Patients with peritoneal dialysis-associated peritonitis (8)	UF and UHPLC-MS/MS	M:49% (I:45.1–55.1%)	
Cefalothin in serum	Craig et al. (1973)	Healthy volunteers (6)	ED and microbiological assay	65.2% ± 4.3%	
	Ohashi et al. (1986)	Patients with cirrhosis (12) or hepatitis (8) and normal volunteers (12)	UF and microbiological assay	Cirrhotic patients: 73.5 ± 4.01% Hepatitis patients: 75.0 ± 3.0%	
				Control group: 75.9 ± 3.4%	
Cefapirin in pericardial fluid	Green et al. (1981)	Pediatric patients (2–17 years) (32)	UF and microbiological assay	36.7% ± 10.7% (<1 h after the administration in pericard eff.)	
Second generation cephalosporins					
Cefamandole in plasma	Berkhout et al. (2003)	Patients treated with cefamandole (10)	ED and HPLC-UV	M:68% (R:62–75%)	Linear
Cefonicid in wound drainage	Swanson et al. (1991)	Patients undergoing oncologic head and neck surgery (6)	UF and HPLC-UV	85%	
Cefonicid in serum	Ackerman et al. (1988)	Patients undergoing hip reconstructive procedures (10) and controls (4)	UF and HPLC	Patients: 88% ± 5.41% after 0.5 h, 92.97% ± 6.56% after 4 h and 96.54% ± 1.44% after 12 h	Non-linear
				Controls: 93.02% ± 1% after 0.5 h and 96.04% ± 1.79% after 4 h	
				Overall: 92.0% ± 16%	
	Dudley et al. (1986)	Healthy volunteers (6)	UF and microbiological assay	82.4 ± 6.1% immediately after administration and 98% for total serum concentrations < 100 ug/ml	Non-linear
	Swanson et al. (1991)	Patients undergoing oncologic head and neck surgery (59)	UF and HPLC-UV	89%	
	Trang et al. (1989)	Geriatric hospitalized male subjects with urinary tract infection (10) and young non-hospitalized male subjects as controls (10)	UF and HPLC-UV	Geriatric: 85.3 ± 8.5% after 0.5h, 96.4 ± 1.6% after 4h and 96.9 ± 1.6% after 12 h	Non-linear
				Young: 93% ± 1% after 0.5h, 96.9% ± 0.3% after 4 h	
Ceforanide in serum	DiPiro et al. (1985)	Hospitalized adult patients scheduled for cholecystectomies (15)	UF and HPLC-UV	87.9%	Linear
Cefotetan in plasma	Yates et al. (1983)	Healthy male Caucasian volunteers (10)	ED and HPLC-UV/microbiological assay	88% (R:78–91%)	
Cefotetan in serum	Carver et al. (1989)	Healthy volunteers (6)	UF and microbiological assay	85% ± 4.2%	Linear
Cefoxitin in blister fluid	Wise et al. (1980)	Healthy volunteers	UF and microbiological assay	59%	
Cefoxitin in plasma	Garcia et al. (1979a)	Patients with terminal renal impairment and undergoing 6 h HD sessions (10)	Method for separation bound/free not known and microbiological assay	41.46% (R:31.03–50%) (during HD)	
	Garcia et al. (1979b)	Patients with normal renal function (10) and varying degrees of renal impairment (13) and patients with terminal impairment (7)	UF and microbiological assay	73.22% normal renal function, decreasing with CrCl to 20–40% at CrCl of 20 ml/min	
Cefoxitin in serum	Carver et al. (1989)	Healthy volunteers (6)	UF and microbiological assay	52% ± 2.8%	Linear
Cefuroxime in blister fluid	Wise et al. (1980)	Healthy male volunteers (6)	UF and microbiological assay	34%	
Cefuroxime in plasma	Aalbers et al. (2015)	Patients undergoing CABG (21)	UF and HPLC	27.5% ± 5.0%	
	Mand'ák et al. (2007)	Patients undergoing CABG (9)	UF and HPLC-UV-PDA	16.3%	
	van Raaij et al. (2020)	Critically ill patients with hypoalbuminemia and renal failure (11)	UF and UPLC-MS/MS	M:24.58% (R:0.25–72.64%)	
	Verhagen et al. (1994)	Healthy male volunteers (6)	ED and HLPLC	17.2% ± 4.2%	
Cefuroxime in serum	Foord, (1976)	Normal male subjects (5)	UF and microbiological assay/HPLC	33% ± 5.7%	
Third generation cephalosporins					
Cefixime in serum	Faulkner et al. (1988)	Young (12) and elderly subjects (12)	ED and HPLC-UV	67%	
Cefmenoxime in serum	Reitberg et al. (1984)	Critical patients with gram-negative pneumonia (20)	ED and HPLC-UV	43.5% ± 13.0%	Linear
Cefodizime in bronchial secretion	Scaglione et al. (1997a)	Patients with acute exacerbation of chronic bronchitis (13)	UF and HPLC-UV	68.1% at 2 h	
				68.8% at 4 h	
				70.2% at 8 h	
				73.1% at 12 h	
Cefodizime in skin blister fluid	Schafer-Korting et al. (1986)	Healthy male volunteers (6)	UF and microbiological assay	61.6% ± 2.7%	
Cefodizime in serum	Scaglione et al. (1997a)	Patients with acute exacerbation of chronic bronchitis (13)	UF and HPLC-UV	81.1% at 2 h	
				81.3% at 4 h	
				82.9% at 8 h	
				84% at 12 h	
	Scaglione et al. (1997b)	Patients undergoing hip arthroplasty (22)	UF and HPLC-UV	85.45%	
	Schafer-Korting et al. (1986)	Healthy male volunteers (6)	ED and microbiological assay	81%	
Cefoperazone in plasma	Kan et al. (2020)	Pediatric children: newborns (17), infants (10) and children (19)	UF and LC-MS/MS	Overall: 83.3% (R:52–91.9%)	
				Newborn: 74.5% ± 9.1%	
				Infants: 82.2% ± 7.1%	
				Children: 87.5% ± 3.2%	
				Lower albumin: 74.5% ± 9.7%	
				Higher albumin: 84.4% ± 6.7%	
	Lam et al. (1988)	Healthy male volunteers (6)	UF and HPLC-UV	91.5% ± 2.0%	
	Rao et al. (2020)	Critically ill patients (8)	UF and LC-MS/MS	R:79.74–99.14%	
Cefoperazone in serum	Kalman et al. (1992)	Healthy male volunteers (6)	UC and HPLC-UV	Ca 90%	
Cefotaxime in pleural exudate	Scaglione et al. (1990)	Patients with pleural empyema treated by intercostal drainage (12)	ED and microbiological assay	7.63%	
Cefotaxime in peritoneal fluid	Seguin et al. (2009)	Critically ill patients with secondary peritonitis (11)	UF and HPLC-UV	12.9% on day 3	
Cefotaxime in plasma	Dahyot-Fizelier et al. (2013)	Patients with acute brain injury (5)	UF and HPLC-UV	40.6% (R:32–52.6%)	Linear
	Seguin et al. (2009)	Critically ill patients with secondary peritonitis (11)	UF and HPLC-UV	18.2% ± 5.9% on day 2 and 17.4% ± 7.7% on day 3	
Cefotaxime in serum	Aardema et al. (2020)	Critically ill patients (59)	UF and LC-MS/MS	Intermittent: M:29.45% (I:25–34.78%)	
				Continuous: M:29.86% (I:25.83–33.71%)	
	Scaglione et al. (1990)	Patients with pleural empyema treated by intercostal drainage (12)	ED and microbiological assay	9.93%	Linear
Cefpiramide in plasma	Demotes-Mainard et al. (1991)	Patients with cirrhosis and ascites (11) and healthy male volunteers (11)	UF and HPLC	Patients: 89.6% ± 9.5%	Non-linear
				Volunteers: 98.1% ± 0.3%	
	Demotes-Mainard et al. (1994)	Patients with cholestasis (8)	UF and HPLC-UV	77% ± 0.13%	
Cefpiramide in serum	Conte, (1987)	Healthy volunteers (10), patients with normal or renal impairment (10) and patients with chronic HD (10)	UF and HPLC	Volunteers: 92.2% ± 1.4%–99.3% ± 0.8%	Non-linear
				Patients with normal or renal impairment: 91.1% ± 1.8%–98.2% ± 0.8%	
				Patients with chronic HD: 88.5% ± 7.1%–94.9% ± 4.8%	
Ceftazidime in plasma	Berkhout et al. (2003)	Patients treated with ceftazidime (5)	ED and LC-UV	0%	
	Isla et al. (2007)	Male patients in the ICU undergoing CVVH or CVVHD (4)	UF and HPLC-UV	14% ± 8%	
	Lam et al. (1988)	Healthy male volunteers (6)	UF and HPLC-UV	21.0% ± 6.0%	
	Matzke et al. (2000b)	Patients with end-stage renal disease receiving conventional maintenance HD (8)	UF and HPLC-UV	17% ± 6% (R:10–25%)	
	Schieser et al. (2021)	ICU patients (7)	UF and HPLC-UV	0%	
Ceftazidime in serum	Van Dalen et al. (1986)	Patients in an ICU with varying degrees of renal function, including patients on regular HD (20)	UF and HPLC-UV	<8% except in 4 patients in whom it was 20–30% (R:0–31%)	
Ceftizoxime in cord serum	Fortunato et al. (1993)	25 samples of cord serum	UF and HPLC-UV	21.9% ± 0.04%	
Ceftizoxime in maternal serum	Fortunato et al. (1993)	25 samples of pregnant women	UF and HPLC-UV	57.8% ± 0.04%	
Ceftriaxone in cerebrospinal fluid	Hoshino et al. (2010)	Pediatric patients with meningitis (2 months-5 years) (12)	UF and microbiological assay	18.8% ± 6.21%	
Ceftriaxone in bronchial secretion	Scaglione et al. (1997a)	Patients with acute exacerbation of chronic bronchitis (12)	UF and HPLC-UV	80% at 2 h	
				78.4% at 4 h	
				79.7% at 8 h	
				80.8% at 12 h	
Ceftriaxone in pleural exudate	Scaglione et al. (1990)	Patients with pleural empyema treated by intercostal drainage (12)	ED and microbiological assay	60.56%	
Ceftriaxone in plasma	Bos et al. (2018)	Severely ill adults in sub-Saharan African patients (88)	UF and HPLC-MS	M:81% (I:71–87%)	Non-linear
	Ebisch et al. (2020)	Critically ill patient requiring CVVH (1)	UF and UPLC-MS/MS	M:32.3% (I:26.3–41.1%)	
	Gijsen et al. (2021)	Critically ill patients with severe community-acquired pneumonia (31)	ED and UHPLC-MS/MS	M:83% (R:50–94.7%)	Non-linear
				At trough M:87% (R:78–94.7%)	
	Grégoire et al. (2019)	Patients with suspected bacterial meningitis (153)	UF and HPLC-UV	M:92.43% (R:50.7–98.39%)	Non-linear
	Hartman et al. (2021)	Critically ill children 0.1–16.7 years, median 2.5 (43)	UF and HPLC-MS	M:86.4% (R:29.7–92.4%)	Non-linear
	Herrera-Hidalgo et al. (2020)	Healthy volunteers (12)	UF and LC-MS/MS	A 93.38% ± 2.15%	Non-linear
				B 91.40% ± 5.65% (two different doses)	
	Kan et al. (2021)	Children (92)	UF and HPLC-UV	88.1% ± 6.3% (R:60–95.2%)	Non-linear
	Luderer et al. (1984)	Young (8) and elderly (8)	ED and HPLC-UV	Young: 83% ± 2.4% at 0.5 h sample and 88.6% ± 1.2% at 4 h sample	
				Old: 77.8% ± 7.0% at 0.5 h sample and 85.4% ± 3.5% at 4 h sample	
	Matzke et al. (2000b)	Patients receiving HD (8)	UF and HPLC-UV	43 ± 15% (R:13–92%)	Non-linear
	Mimoz et al. (2000)	Patients with hydroxyethyl starch-induced hypoalbuminemia (11) and matched healthy volunteers as controls (11)	UF and HPLC	Patients: M:82% (R:75–86%)	
				Volunteers: M:90% (R:74–94%)	
	Neves et al. (2018)	Healthy adult volunteers (12)	Method for separation bound/free not known and LC-MS/MS	Eltrombopag 0 mg: 91.1% (86.3–94%)	
				Eltrombopag 25 mg: 89.1% (86.8–91.3%)	
				Eltrombopag 50 mg: 90% (87–92.9%)	
	Popick et al. (1987)	Healthy subjects (12)	ED and HPLC-UV	R:82.2–89.0%	Non-linear
	Schleibinger et al. (2015)	ICU patients (17)	UF and HPLC-UV	M:67% (I:54.5–79.8%)	
	Stoeckel et al. (1984)	Normal subjects (8) and subjects with various degrees of chronic liver damage (alcoholic fatty liver, cirrhosis without ascites and cirrhosis with ascites (15)	ED and HPLC	Normal: 95% ± 0.8%	Non-linear
				Fatty liver: 92.9% ± 2.1%	
				Cirrhosis without ascites: 90.9% ± 2.0%	
				Cirrhosis with ascites: 83.9% ± 6.1%	
	Toth et al. (1991)	Adult orthotopic liver transplant patients (7)	ED and HPLC-UV	R:44–95%	Non-linear
	Tsai et al. (2016)	Critically ill Australian Indigenous patients with severe sepsis (5)	UF and UHPLC-MS/MS	R:57–86%	Non-linear
	Ulldemolins et al. (2021)	Patients with septic shock and hypoalbuminemia receiving CVVH (8)	UF and LC-MS/MS	56%	
	Wong et al. (2018)	Critically ill patients (n.a.)	UF and HPLC-UV	R:83–95%	
Ceftriaxone in serum	Bourget et al. (1993)	Pregnant women with chorioamnionitis or pyelonephritis (9)	UF and HPLC-UV	92.58 ± 14.2%	Non-linear
	Fukumoto et al. (2009)	Pediatric patients with pneumonia (8)	UF and HPLC-UV	∼80–90% for mid-concentrations	Non-linear
	Heinemeyer et al. (1990)	Surgical intensive care patients with a bacterial infection of the bronchial tract with normal renal function (6) or with acute renal failure (5)	ED and HPLC	Normal renal function: R:85.5–91.5%	
				Acute renal failure: R:70.9–83.6%	
	Hoshino et al. (2010)	Pediatric patients with meningitis (2 months-5 years) (7)	UF and microbiological assay	81.8% ± 8.35%	
	Meenks et al. (2021)	ICU patients with sepsis (5)	UF and UPLC-MS/MC	M:70.9% (I:47.8–84.8%)	
	Scaglione et al. (1997a)	Patients with acute exacerbation of chronic bronchitis (12)	UF and HPLC-UV	92.5% at 2 h	
				93% at 4 h	
				93.6% at 8 h	
				94% at 12 h	
				93.8% at 24 h	
	Scaglione et al. (1997b)	Patients undergoing hip arthroplasty (20)	UF and HPLC-UV	79.16%	
	Scaglione et al. (1990)	Patients with pleural empyema treated by intercostal drainage (12)	ED and microbiological assay	70.17%	Linear
Fourth generation cephalosporins					
Cefepime in plasma	Al-Shaer et al. (2020)	Patients in University of Florida Health Shands Hospital where the physicians requested drug concentrations to allow the optimization of treatment (36)	UF and LC-MS	M:39% (R:29–61%)	
	Isla et al. (2005)	Male patients undergoing CVVH or CVVHD (2)	UF and HPLC-UV	21% ± 9%	
Cefepime in serum	Kalman et al. (1992)	Healthy male volunteers (6)	UC and HPLC-UV	Ca 20%	
Cefluprenam (E1077) in plasma	Nakashima et al. (1994)	Healthy male volunteers (36)	UF and microbiological assay/HPLC-UV	14.5% ± 2.9%	Linear
Fifth generation cephalosporins					
Ceftobiprole in plasma	Barbour et al. (2009)	Healthy volunteers (15)	UF and HPLC-MS/MS	21.7% ± 6.6%	
Ceftolozane in plasma	Kratzer et al. (2019)	Healthy volunteers (n.a.)	UF and HPLC-UV	6.3% ± 2.0%	

CABG, coronary artery bypass graft; CPB, cardiopulmonary bypass; CVVHD, continuous venovenous hemodialysis; CVVH, continuous venovenous hemofiltration; DHCA, deep hypothermic circulatory arrest; ED, equilibrium dialysis; ECMO, extracorporeal membrane oxygenation; HD, hemodialysis; HPLC, high-performance liquid chromatography; HPLC-MS, high-performance liquid chromatography-mass spectrometry; HPLC-MS/MS, high-performance liquid chromatography with tandem mass spectrometry; HPLC-UV, high-performance liquid chromatography with ultraviolet detection; HPLC-UV-PDA, high-performance liquid chromatography with ultraviolet photodiode-array detection; ICU, intensive care unit; I, interquartile range; LC-MS/MS, liquid chromatography with tandem mass spectrometry, M, median; NICU, neonatal intensive care unit; R, range; UC, ultracentrifugation; UF, ultrafiltration, UHPLC-MS/MS, ultra-high-performance liquid chromatography with tandem mass spectrometry.

The included studies represented the protein binding of 23 different cephalosporins in 11 different body fluids (N = 117): plasma (N = 62), serum (N = 38), pericardial fluid (N = 4), blister fluid (N = 3) wound exudate (N = 2), bronchial secretion (N = 2), pleural exudate (N = 2), dialysate (N = 1), peritoneal fluid (N = 1), cerebrospinal fluid (N = 1), and cord serum (N = 1). Some studies reported the protein binding in more than one fluid. The protein binding of cefazolin and ceftriaxone was studied most extensively. Protein binding was mostly determined in blood serum or blood plasma compared to other body.

#### 3.2.3 Protein Binding in Plasma and Serum

**FIGURE 3:**
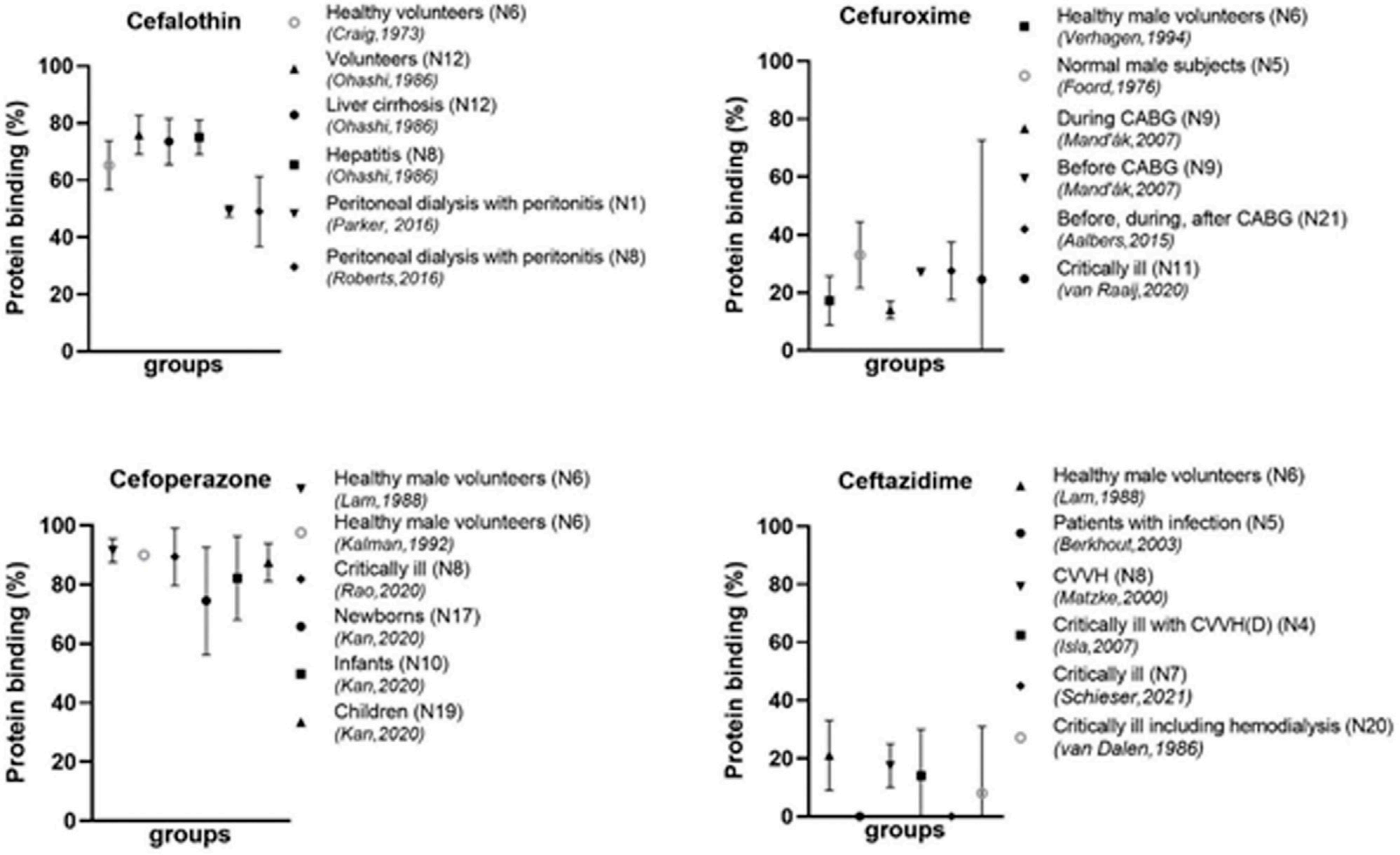
Protein binding of different cephalosporins in different populations N, number of participants; CABG, coronary artery bypass graft; CVVHD, continuous venovenous hemodialysis; CVVH, continuous venovenous hemofiltration; ECMO, extracorporeal membrane oxygenation.

Overall, the degree in protein binding is variable between the different cephalosporins (see examples in Figure 3). Even between studies of an individual cephalosporin, there is considerable variability. As can be seen in Table 1, several cephalosporins are highly protein bound, such as cefonicid (mean 82%–98%, four studies) (Dudley et al., 1986; Ackerman et al., 1988; Trang et al., 1989; Swanson et al., 1991) and cefoperazone in adults (mean 89%–92%, four studies) (Lam et al., 1988; Kalman et al., 1992; Kan et al., 2020; Rao et al., 2020). Also, ceftriaxone is generally highly bound to proteins, although there is a large variability between studies. Some cephalosporins show a consistently low protein binding, such as cefuroxime (mean 16%–33%, five studies) (Foord, 1976; Verhagen et al., 1994; Mand'ák et al., 2007; Aalbers et al., 2015; van Raaij et al., 2020), cefotaxime (mean 8%–41%, four studies) (Scaglione et al., 1990; Seguin et al., 2009; Dahyot-Fizelier et al., 2013; Aardema et al., 2020) and ceftazidime (mean 0%–21%, six studies) (Van Dalen et al., 1986; Lam et al., 1988; Matzke et al., 2000a; Berkhout et al., 2003; Isla et al., 2007; Schieser et al., 2021).

**FIGURE 4:**
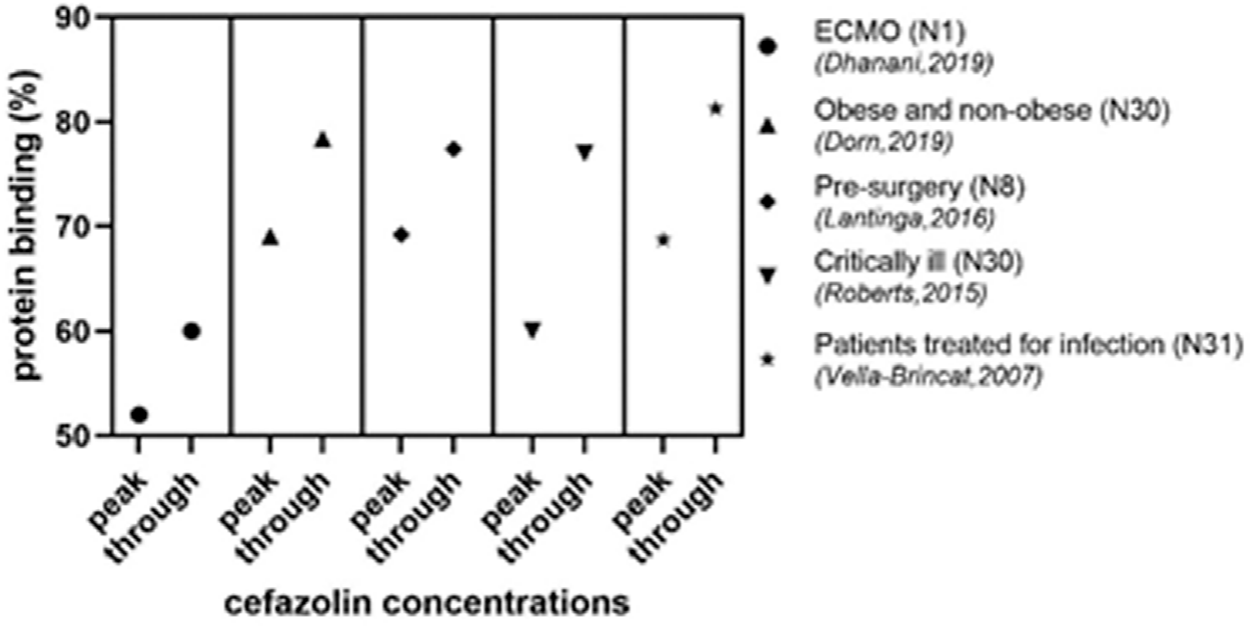
Peak and trough concentrations of cefazolin in different populations N, number of participants; ECMO, extracorporeal membrane oxygenation.

In some studies the pattern of protein binding was mentioned as well. For the concentration range as achieved in clinical patients, the protein binding was found to be linear for ceforanide (DiPiro et al., 1985), cefamandole (Berkhout et al., 2003), cefmenoxime (Reitberg et al., 1984), cefotaxime (Scaglione et al., 1990; Dahyot-Fizelier et al., 2013), cefluprenam (Nakashima et al., 1994), cefotetan (Carver et al., 1989), and cefoxitin (Carver et al., 1989). However, for some cephalosporins a typical non-linear pattern was found. A clear example hereof is cefazolin. As shown in Figure 4, the protein binding for the peak-concentrations is consistently lower in five studies as compared to the through-concentrations (Vella-Brincat et al., 2007; Roberts et al., 2015; Lantinga et al., 2016; Dhanani et al., 2019; Dorn et al., 2019). This non-linear behavior was shown for different categories of patients. Other cephalosporins with a non-linear pattern are cefonicid (Dudley et al., 1986; Ackerman et al., 1988; Trang et al., 1989), cefpiramide (Conte, 1987; Demotes-Mainard et al., 1991), and ceftriaxone (Stoeckel et al., 1984; Popick et al., 1987; Toth et al., 1991; Bourget et al., 1993; Matzke et al., 2000b; Fukumoto et al., 2009; Tsai et al., 2016; Bos et al., 2018; Grégoire et al., 2019; Herrera-Hidalgo et al., 2020; Gijsen et al., 2021; Hartman et al., 2021; Kan et al., 2021). An atypical non-linear pattern was not found for any of the cephalosporins.

#### 3.2.4 Factors Potentially Influencing Protein Binding

An important factor determining the degree of protein binding is the serum albumin level. A low level of serum albumin is expected to result in a lower protein binding. Correlations between the albumin concentrations and protein binding were seen for cefazolin, ceftizoxime, ceftriaxone, and cefoperazone (Ohashi et al., 1986; Fortunato et al., 1993; Allegaert et al., 2009; Himebauch et al., 2014; Schleibinger et al., 2015; de Cock et al., 2017; Asada et al., 2018; Kan et al., 2020; Booke et al., 2021; Hartman et al., 2021). However, the studies of Toth et al. (1991) and Tsai et al. (2016) showed no correlation between the albumin concentration and the protein binding of ceftriaxone. For cefonicid and cefmonoxime, no correlation was seen between albumin concentrations and protein binding (Reitberg et al., 1984; Trang et al., 1989).

Several medical characteristics are known to be associated with a low serum albumin level and are therefore expected to result in lower degrees of protein binding. Examples will be described.

Patients on dialysis are known to have low albumin concentrations. This hypoalbuminemia is multifactorial and caused by malnutrition, exogenous albumin loss, and volume expansion (Yeun and Kaysen, 1998). Other factors such as the type of dialyses, surface area, type of membrane, blood flow and dialysate flow might also influence protein binding (Garcia et al., 1979a). This is in line with the results found for the different cephalosporins. Protein binding in several patients on peritoneal dialysis with a peritonitis has been described. Mean or median values for the protein binding of cefazolin was found to be between 64% and 82% (Roberts et al., 2015; Parker et al., 2016; Roberts et al., 2016), while in healthy volunteers these values were higher 84%–89% (Craig et al., 1973; Ohashi et al., 1986). In a 25-year old patient on extracorporeal membrane oxygenation (ECMO) the protein binding was even lower (52%–60%) (Dhanani et al., 2019). For cefalothin, the protein binding for patients on peritoneal dialysis with a peritonitis was found to be 47%–51% (Parker et al., 2016; Roberts et al., 2016) as compared to healthy volunteers of mean 65.2% (Craig et al., 1973) (Figure 3). For patients on hemodialysis, a lower degree in protein binding has also been shown. For example, protein binding for cefoxitin was mean 41.5% in patients on hemodialysis (Garcia et al., 1979a), compared healthy volunteers (mean 52%) (Carver et al., 1989). The same applies to ceftriaxone [mean 43% for dialysis patients (Matzke et al., 2000b) and 90%–93% for healthy volunteers (Mimoz et al., 2000; Neves et al., 2018; Herrera-Hidalgo et al., 2020)].

Critically ill patients are another important group. For several cephalosporins low protein binding has been described in this patient category. Cefazolin protein binding in critically ill adults was found to be 60%–77% (Roberts et al., 2015). This is lower compared to healthy volunteers (84%–89%) (Craig et al., 1973; Ohashi et al., 1986). In critically ill patients on ECMO its protein binding was even lower (mean 51%) (Booke et al., 2021). Also for ceftriaxone several studies have been performed in critically ill adults, all resulting in a lower protein binding compared to healthy volunteers (Schleibinger et al., 2015; Tsai et al., 2016; Bos et al., 2018; Gijsen et al., 2021; Meenks et al., 2021; Ulldemolins et al., 2021). However, also within this category of patients the degree of protein binding varies considerably, with values from 50% to 94%. Tsai et al. (2016) concluded that the protein binding of ceftriaxone was not correlated with albumin, but with hyperbilirubinemia and diabetes mellitus.

Patients with liver cirrhosis might also have lower values for the protein binding of cephalosporins. This could be due to less efficient removal of cumulated endogenous binding inhibitors in patients with cirrhosis and ascites (Stoeckel et al., 1984). This was confirmed for cefazolin, which was bound for mean 72.3% in cirrhotic patients (Ohashi et al., 1986). Also for ceftriaxone protein binding was lower (83.9% vs. 90%–93% in healthy volunteers) (Stoeckel et al., 1984; Mimoz et al., 2000; Neves et al., 2018; Herrera-Hidalgo et al., 2020) as well as for cefpiramide (89.6 vs. 98.1 in healthy volunteers) (Demotes-Mainard et al., 1991). For cefalothin there was not a significant difference in protein binding between patients with cirrhosis (73.4%) and controls (75.9%) (Ohashi et al., 1986).

Pregnancy is also described as to be associated with a low albumin level. Limited data are available on the protein binding during pregnancy. Allegaert et al. (2009) showed a reduced protein binding of 75% in 30 pregnant women treated with cefazolin as compared to 84%–89% protein binding in healthy volunteers (Craig et al., 1973; Ohashi et al., 1986; Allegaert et al., 2009). However, in a study with ceftriaxone the protein binding was not reduced and found to be 92% in pregnant women with chorioamnionitis or pyelonephritis (Bourget et al., 1993).

Cardiopulmonary bypass (CPB) led to differences in protein binding during the procedure (Hollis et al., 2015; de Cock et al., 2017; Asada et al., 2018). Cefazolin protein binding was shown to be low (53%–55%) during CBP (Hollis et al., 2015; Asada et al., 2018). One study compared the binding during CBP with the binding preoperative in the same group of patients and found different values of 55% during CBP and 79% pre-CBP (Asada et al., 2018). For cefuroxime the data were not consistent. Mand’ák et al. (2007) showed a low protein binding of 11%–17% during the procedure as compared to 27% before the onset of the study, while another study found no difference in protein binding due to CBP (Aalbers et al., 2015).

Patients with viral, bacterial or fungal infections might also have hypoalbuminemia (Wiedermann, 2021). Studies on protein binding on cephalosporins in patients with infections without other causes of hypoalbuminemia were not frequently performed. For cefazolin two studies were performed in infectious patients. Protein binding in patients treated with cefazolin for an infection was 81.3% for through-concentrations and 68.7% for the peak-concentrations (Vella-Brincat et al., 2007). This was not different from a study in obese and non-obese patients reporting a median protein binding of 79% (Van Kralingen et al., 2011). Also, in patients with an acute exacerbation of chronic bronchitis there was not a relevant reduction in protein binding as compared to volunteers for both cefodizime and ceftriaxone (Scaglione et al., 1997a; Herrera-Hidalgo et al., 2020). In patients with hepatitis, cefazolin protein binding was 84.7% versus 88.6% in controls (Ohashi et al., 1986). These studies indicate that a profound reduction in protein binding caused by infection itself has not been proven yet.

#### 3.2.5 Newborns and Children

Many physiological changes take place during development from newborn to adult. Therefore, differences in protein binding as compared to adults might be expected. Unfortunately, limited data are available for newborns and children. From the available data it becomes clear that protein binding might be reduced in children, especially newborns (Deguchi et al., 1988; Kan et al., 2020). A study in children from 2 months to 4 years of age treated with ceftriaxone for meningitis (Hoshino et al., 2010) can be compared to data in adults with suspected in meningitis (Grégoire et al., 2019). In children the protein binding was 81.8% ± 8.4% and in adults the median value was higher (92.4%), however the range in adults was larger (51%–98%). The cefazolin protein binding in neonates in Intensive Care Unit (ICU) was found to be lower (49%) (Deguchi et al., 1988), while values for adults in the ICU were around 60%–77% (Roberts et al., 2015). A study on cefoperazone compared three age groups: newborns (30.1–42.3 weeks), infants (0.1–2.0 years) and children (2.2–9.0 years) (Kan et al., 2020). They found the lowest protein binding in newborns (74.5% ± 9.1%), and higher protein binding for infants (82.2% ± 7.1%) and even higher values for children (87.5% ± 3.2%) (Figure 3). Differences were also correlated to the levels of albumin.

One study determined the protein binding in cord blood. Lower protein binding in cord serum than in maternal serum was seen for ceftizoxime (21.9% vs. 57.8%). As arterial cord blood originates from the fetus, the lower binding of combined arterial/venous cord blood is in line with the lower protein binding found in newborns and the lower protein binding as described in pregnant women. It could also be due to difference in protein milieu or high level of bilirubin in cord serum competing for binding sites and an increase in available albumin in maternal serum (Fortunato et al., 1993).

#### 3.2.6 Other Factors

The influence of several other factors has been studied as well, but the influence on protein binding was not present or not relevant. For cefazolin several studies investigated whether there was a differences between (morbidly) obese and non—obese patients (Van Kralingen et al., 2011; Hites et al., 2016; Palma et al., 2018; Dorn et al., 2019). It was concluded that obesity did not influence protein binding. The influence of various degrees of renal impairment (with the exception of dialysis) did not influence protein binding (Craig et al., 1973; Brodwall et al., 1977). Small differences are possibly related to differences in protein levels rather than renal function (Garcia et al., 1979b). For cefazolin and cefalothin has been shown that hepatitis did not have an effect on protein binding (Ohashi et al., 1986). Patients undergoing elective surgery are also studied and found to be relatively similar to healthy volunteers (Scaglione et al., 1997b; Douglas et al., 2011). Age, as the only factor, will likely not influence protein binding (Faulkner et al., 1988), however small differences might be present and they might be caused by a lower protein level in the elderly (Luderer et al., 1984).

#### 3.2.7 Protein Binding in Other Body Fluids

##### 3.2.7.1 Pericardial Fluid

The protein binding in pericardial fluid was determined for cefazolin, cefradine, cefalothin, and cefapirin. The protein binding of cefazolin in pericardial fluid in patients undergoing heart surgery was slightly equal to binding in serum of healthy volunteers (83.8% and 83.9%/88.6% respectively) (Craig et al., 1973; Nightingale et al., 1980; Ohashi et al., 1986). For cefradine, the protein binding in pericardial fluid in patients undergoing heart surgery was <10%, which was lower than that of serum seen in a patient with continuous ambulatory peritoneal dialysis for end-stage renal failure with and were therefore expected to be lower than in healthy volunteers (Nightingale et al., 1980; Martea et al., 1987). The protein binding of cefalothin in pericardial fluid of pediatric patients (65.2%) was lower than that in serum of healthy adult volunteers (83.9%/88.6%) (Craig et al., 1973; Green et al., 1981; Ohashi et al., 1986). The protein binding of cefapirin in pericardial fluid of pediatric patients was 36.7% (Green et al., 1981). This could not be compared to the protein binding of blood since we could not include a study which determined the protein binding in blood.

##### 3.2.7.2 Blister Fluid

Whether the protein binding in blister fluid is lower, equal to or higher than in blood differs per cephasporin. For cefoxitin, cefuroxime and cefodizime the protein binding in blister fluid was determined.

The protein binding of cefoxitin in blister fluid of healthy volunteers might be slightly higher than that in serum (59% vs. 52%) (Wise et al., 1980; Carver et al., 1989). However, the study of Garcia et al. (1979b) showed a protein binding of 73.22% in plasma which was much higher than that of Carver et al. (1989) (52%). The protein binding of cefuroxime in blister fluid was 34% (Wise et al., 1980) and this is equal to that in serum of healthy males (33%) (Foord, 1976), but higher compared to another study with healthy volunteers (17.2%) (Verhagen et al., 1994). The protein binding of cefodizime in skin blister fluid was significantly lower than in serum of the same healthy subjects (61.6% vs. 81%) (Schafer-Korting et al., 1986).

##### 3.2.7.3 Bronchial Secretion and Pleural Exudate

The protein binding of cefodizime in bronchial secretion (68.1%–73.1%) was lower than that in serum (81.1%–84%) of the same subjects. This was also the case for ceftriaxone 78.4%–80.8% (bronchial secretion) vs. 92.5%–94% (serum) (Scaglione et al., 1997a). One study determined the protein binding of cefotaxime and ceftriaxone in pleural exudate. The protein binding was lower than in serum of the same subjects (Scaglione et al., 1990). For cefotaxime the difference was smaller (7.64% vs. 9.93%) than for ceftriaxone (60.56% vs. 70.17%). However, the protein binding of cefotaxime in serum and plasma differed in various populations (Scaglione et al., 1990; Seguin et al., 2009; Dahyot-Fizelier et al., 2013; Aardema et al., 2020).

##### 3.2.7.4 Wound Exudate

Protein binding in wound exudate was lower than in serum. The protein binding of cefazolin in wound exudate in burn and trauma patients was significantly lower than that in serum of healthy volunteers (44.6% vs. 83.9%/88.6%) (Craig et al., 1973; Ohashi et al., 1986; Rowan et al., 2017). There was also a potential lower protein binding for cefonicid seen in wound drainage of oncologic patients undergoing head and neck surgery compared to their protein binding in serum (85% vs. 89%) (Swanson et al., 1991). However, protein binding in serum differs from other studies which determined protein binding in healthy volunteers (Dudley et al., 1986; Ackerman et al., 1988; Trang et al., 1989). This could be due to the advanced age, nutritional status, and illness of the oncologic patients (Swanson et al., 1991).

##### 3.2.7.5 Cerebrospinal Fluid

The protein binding of ceftriaxone in cerebrospinal fluid (18.8% ± 6.21%) was significantly lower than in serum/plasma (ranging from 32.3% to 95%) (Toth et al., 1991; Hoshino et al., 2010; Ebisch et al., 2020).

##### 3.2.7.6 Dialysate and Peritoneal Fluid

The protein binding of cefradine in dialysate from peritoneal dialysis was significantly lower than that in plasma of a patient with continuous ambulatory peritoneal dialysis for end-stage renal failure with peritonitis (6.1% vs. 29.1%) (Martea et al., 1987).

The protein binding of cefotaxime in peritoneal fluid was lower (12.9%) than that in plasma in critically ill patients with secondary peritonitis (17.4%) (Seguin et al., 2009). However, this protein binding in serum is lower than other studies (Dahyot-Fizelier et al., 2013; Aardema et al., 2020).

## 4 Discussion

### 4.1 Summary of Main Findings

In this systematic review, data on protein binding of cephalosporins in human fluids were reviewed. Most studies analyzed protein binding in serum or plasma and only a few in other body fluids, such as wound exudate or pericardial fluid.

For serum and plasma, the degree of protein binding was variable. As expected, there was a large variability between the different cephalosporins. Even for a specific cephalosporin, the protein binding differs between different categories of patients and even within the categories there was a considerable amount of variability. For ceftriaxone, for example, a group of eight patients on hemodialysis displayed a mean protein binding of 43% (Matzke et al., 2000b), which was considerably lower compared to healthy volunteers (mean/median 90%–95%) (Stoeckel et al., 1984; Neves et al., 2018; Herrera-Hidalgo et al., 2020). However, even within the group of dialysis patients the protein binding ranged approximately from 13% to 73% (Matzke et al., 2000b). This shows that based on the mean or median of the groups it is possible to identify categories of patients with expected lower protein binding, but on individual level the percentage is still unpredictable.

Several patient characteristics were identified with overall lower protein binding compared to healthy volunteers. This is mainly caused by a concomitant hypoalbuminemia. These characteristics include critically ill patients, patients on (all types) of dialysis and during a CPB (Garcia et al., 1979a; Mand'ák et al., 2007; Parker et al., 2016; Bos et al., 2018; Aardema et al., 2020; Ebisch et al., 2020). Although studies in children are scarce, available data suggests that protein binding might be lower in children compared to adults, especially in newborns (Kan et al., 2020). Comparison between the different categories of patients is sometimes complicated by the fact that multiple factors are present at the same time, such as a critically ill patients on dialysis in a malnutrition state. These findings are in line with current reports in the literature (Roberts et al., 2013).

Studies on protein binding in other body fluids are scarce. In most studies values in fluids other than blood were lower compared to those in serum or plasma. However, given the low quantity of data and the high variability in serum and plasma, no conclusions can be drawn about other body fluids than serum of plasma.

### 4.2 Limitations

A limitation of the review is that the literature search had to be performed in several steps. The original search was quite broad including all beta-lactams to make sure we did not miss one of the cephalosporins. However, after the systematic search there were missing articles. Protein binding is often reported in studies while it is not the purpose of the specific studies. This is most likely the reason why some articles were missing in the original systematic search. We corrected this manually by searching for studies reporting unbound concentrations of the cephalosporins found in the systematic search as well as checking the references lists of the included articles. By using this method we were able to add 26 articles to the selection.

Except from differences between patients or patient groups, also other factors might contribute to the variability in protein binding as found in our review. One of these factors is the non-linear protein binding, as found for f.e. ceftriaxone. Despite the different percentages of protein binding dependent on the concentration, many studies only report a single percentage as mean with variability. Part of the variability might therefore be explained by the total concentration and thereby also sampling time in relation to the dose. Another factor might be the method used to determine the protein binding. Most of the studies used equilibrium dialysis (ED) or ultrafiltration (UF) as method for the separation of free drug from the protein bound. ED is accepted as the “golden standard” to determine the free fraction of a drug. This method is based on the diffusion of free drug through the a permeable membrane until there is an equilibrium between both compartments. The protein bound fraction cannot pass this membrane. ED is often performed at 37°C to simulate *in vivo* conditions. ED is a time consuming procedure because of the long equilibrium time (3–48 h for conventional equilibrium dialysis, 2–6 h for rapid ED). UF is much quicker. It separates bound and unbound drug from each other through a filter membrane with centrifugal force. Disadvantages are that there is non-specific binding to this membrane and temperature cannot be controlled (de Boer and Meijering, 2019). The non-specific binding makes it extra important that validation was done prior to the analysis.

### 4.3 Clinical Relevance

In this review only studies measuring protein binding after administering the antibiotic to humans were included. So, studies using human samples to determine protein binding after adding antibiotics in the laboratory were excluded. As a result only clinically achievable concentration ranges were included and artificial influences, such as temperature and pH (Hinderling and Hartmann, 2005), were excluded. Interindividual differences can also be determined when using samples instead of adding the drug to a body fluid (Wright et al., 1996). Kratzer et al. (2019) found differences in the protein binding of ceftolozane and tazobactam between *in vitro* and *ex vivo* plasma samples. Also in plasma of dogs differences were seen in in vitro and *ex vivo* samples (Stegemann et al., 2006). However, for voriconazole there were no differences in the protein binding between *in vitro* experiments and samples from patients (Vanstraelen et al., 2014).

A more in-depth evaluation of the data also showed great differences in protein binding, as becomes clear from the following examples. The protein binding of ceftazidime was low with a range of 0%–31% (Van Dalen et al., 1986), while the protein binding of cefpiramide and cefodizim was higher (77%–99.3% and 81%–85.45% respectively) (Schafer-Korting et al., 1986; Conte, 1987; Demotes-Mainard et al., 1994; Scaglione et al., 1997b). When also taking into account different patient populations even more variability was described, such as within critically ill patients. The range in protein binding of ceftriaxone in these patients varied between 26.3% and 95% (Wong et al., 2018; Ebisch et al., 2020). Cefuroxime also showed a great variation in protein binding in critically ill patients (0.25%–72.64%) (van Raaij et al., 2020). As mentioned earlier, patients on hemodialysis showed lower protein binding (8%) for ceftriaxone compared to healthy volunteers (protein binding 90%–95%) (Stoeckel et al., 1984; Matzke et al., 2000b; Neves et al., 2018; Herrera-Hidalgo et al., 2020). This was also seen for cefepime in patients on continuous venovenous hemofiltration and continuous venovenous hemodialysis. Cefepime was 21% bound to proteins in patients the renal replacement therapies, which was lower compared to other patients where cefepime showed a median protein binding of 39% (Isla et al., 2005; Al-Shaer et al., 2020).

The percentage of protein binding is important in clinical practice, since the effectivity of an antibiotic is correlated to the unbound concentration-time profile. The protein binding in individual patients is hardy predictable, especially in patients with multiple characteristics known to influence protein binding. Several characteristics seems to result in a lower protein binding, but the complex interaction between a reduced protein binding and increased drug clearance results in unpredictable concentration-time profiles. The increased clearance at the beginning of the dosing interval might lead to too low unbound concentrations at through levels. To avoid this, it might be beneficial to extent the duration of infusion on cephalosporins with typical non-linear protein binding. An example hereof is cefazolin used as perioperative prophylaxis. Especially during surgery the concentrations needs to remain adequate over the entire dosing interval. Continuous infusion might therefore be beneficial to prevent subtherapeutic concentrations at the end of the dosing interval.

Therapeutic drug monitoring (TDM) is increasingly used, also for the cephalosporins, to guide individual dosing regimen to optimize the exposure. However, most TDM is currently based on total drug concentrations, that are subsequently corrected for the protein binding using a fixed percentage. Considering the fact that most clinical patients have lower protein binding compared to healthy volunteers, the use of a fixed percentage based on studies in healthy volunteers, will result in an increased exposure based on unbound concentrations in severely ill patients as compared to healthy volunteers. Based on the overview in this review it became clear that the variability between patients and for non-linear drugs also with different concentrations, leads to the recommendation to determine unbound concentrations to be used in TDM for individual patients.

### 4.4 Conclusion

A larger variability was found in the protein binding in serum or plasma between different categories of patients as well as within a category of patients, than was usually account for in clinical practice. The differences described in in protein binding in serum or plasma might be of clinical relevance, since only the free fraction is able to enter the extravascular space where most infections are and bound antibiotic is inactive (Craig and Kunin, 1976; Merrikin et al., 1983; Beer et al., 2009). Several characteristics were identified, such as critically ill patients and patients on dialysis, which are prone to having a low protein binding. The typical non-linear pattern of protein binding as found for several cephalosporins even complicates the possibility to predict the unbound concentration-time profile in individual patients even more. Taken all these factors, it is recommended to measure unbound concentrations to perform TDM to optimize antibiotic exposure in individual patients.

Conclusions on protein binding in different body fluids other than blood cannot be drawn due to the paucity of data. More research is needed to determine the relationship between protein binding in blood and protein binding in other body fluids.
