# Supplementary material for: An Overview of the Protein Binding of Cephalosporins in Human Body Fluids: A Systematic Review
Source: Front Pharmacol. 2022 Jun 28;13:900551. doi: 10.3389/fphar.2022.900551 (PMC9274189; doi:10.3389/fphar.2022.900551)
Supplement: Supplementary file 1 [file DataSheet2.pdf]

Drug + fluid	Reference	Title	Population (number of participants)	Dosage	Time point sampling	Sampling method	Analytical method	Protein binding (mean±SD; or as indicated)	Study type
<u>First generation cephalosporins</u>									
**Cefazolin in pericardial fluid**	Nightingale 1980	Effect of Protein Binding on the Penetration of Nonmetabolized Cephalosporins into Atrial Appendage and Pericardial Fluids in Open-Heart Surgical Patients	Patients undergoing coronary artery bypass or cardiac valve replacement (16)	2 g i.v. 30-118 min before the removal of the right atrial appendage	Immediately upon opening the pericardium	n.a.	UF and microbiological assay	83.8% ± 4.1%	Observational study
**Cefazolin in wound exudate**	Rowan 2017	Wound Penetration of Cefazolin, Ciprofloxacin, Piperacillin, Tazobactam, and Vancomycin During Negative Pressure Wound Therapy	Burn and trauma patients with negative pressure wound therapy (8)	Every 8 h	Throughout the 6, 8, or 12 h blood sampling period	n.a.	UF and HPLC-UV	44.6% ± 23.2%	Prospective, observational study
**Cefazolin in plasma**	Allegaert 2009	Cefazolin plasma protein binding saturability during pregnancy	Pregnant women (30)	2 g i.v. over 30 min 1-2 h before surgery	Peak level: soon after i.v. administration<br>Trough levels: 4-8 h after i.v. administration	Venous blood sampling through a peripherally venous catheters	UF and HPLC-UV	M: 75% (R:59-86%)	Observational study
	Asada 2018	Effects of cardiopulmonary bypass on the disposition of	Patients undergoing cardiothoraci	1 g 1 h before surgery and thereafter 1 g every 4 h	Before, 30 min after the start and the end of CPB and at wound closure	Through an arterial catheter	UF and HPLC-UV	Preoperative: M:79%<br>During CPB: M:55%	Prospective observational study

	cefazolin in patients undergoing cardiothoracic surgery	c surgery with CPB (27)						
Booke 2021	Excessive unbound cefazolin concentrations in critically ill patients receiving veno-arterial extracorporeal membrane oxygenation (vaECMO): an observational study	Critically ill patients on ECMO (6)	Continuous infusion 6 g over 24 h	At steady state, daily on day one, two, three and day six after inclusion	Indwelling arterial catheter	UF and HPLC-UV	51% ± 9%	Prospective, observational study
Deguchi 1988	Interindividual Changes in Volume of Distribution of Cefazolin in Newborn Infants and Its Prediction Based on Physiological Pharmacokinetic Concepts	Newborn infants at NICU (20)	30 mg/kg i.v. infusion for 30 min	30, 60, 90, 120, 240, 480, and 720 min after the start of the infusion	Heal puncture	UF and HPLC	49% ± 0.17	Observational
Dhanani 2019	Pharmacokinetics of Total and Unbound Cefazolin during Veno-Arterial Extracorporeal Membrane Oxygenation: A Case Report	25 year old female on ECMO (1)	2 g every 8 h	1, 4 and 8 h after administration	n.a.	UF and HPLC-MS/MS	After 1h: 52% After 4h: 60% After 8h: 60%	Case report

	Dorn 2019	Determination of total or free cefazolin and metronidazole in human plasma or interstitial fluid by HPLC-UV for pharmacokinetic studies in man	Morbidly obese (15) and non-obese patients (15)	2 g i.v. short infusion within 1 h of incision	Predose and 0.5 (end of infusion), 1, 2, 3, 4, 5, 6 and 8 h after administration	n.a.	UF and HPLC-UV	M:69.1% at high total concentration (132 mg/l) and M:78.4% at low total concentrations (14.4 mg/l)	Observational study
	Douglas 2011	Plasma and Tissue Pharmacokinetics of Cefazolin in Patients Undergoing Elective and Semielective Abdominal Aortic Aneurysm Open Repair Surgery	Patients (semi)elective abdominal aortic aneurysm open repair surgery (12)	2 g over 3 min i.v. bolus injection 30-min prior to incision	At 0 min (immediately prior to administration) and then at 3, 10, 30, 60, 90, 180, 300, and 480 min or until at least the conclusion of surgery after administration	n.a.	UF and LC-MS/MS	M:87% (I:74-90%)	Prospective observational pharmacokinetic study
	Lantinga 2016	Hepatic cyst penetration of cefazolin in patients receiving aspiration sclerotherapy	Patients scheduled to undergo percutaneous aspiration sclerotherapy of a symptomatic non-infected, non-neoplastic hepatic cyst (8)	1 g i.v. 1 h before the procedure	Directly after administration, during cyst fluid drainage and after 4 times the expected t1/2 of cefazolin	Through an i.v. cannula at the contralateral side of the cefazolin administration	UF and HPLC-UV	M:74.2% (I:71.8-81.2%) At peak: M:69.2% During cyst drainage: M:73.6% Last sample: M:77.4%	Observational study / extrapolative clinical trial

Naik 2017	Comparative total and unbound pharmacokinetics of cefazolin administered by bolus versus continuous infusion in patients undergoing major surgery: a randomized controlled trial	Patients undergoing urological or multilevel spine surgery (20)	2 g i.v. before surgical incision Intermittent bolus: 2 g every 4h until skin closure Continuous infusion: 500 mg/h until skin closure	5, 30 and 120 min after administration	From an arterial line	Method for separation bound/free not known and UHPLC-MS/MS	69% (R:44–80%)	Prospective open-label observational randomized controlled pharmacokinetic trial
Palma 2018	Fast and sensitive HPLC/UV method for cefazolin quantification in plasma and subcutaneous tissuemicrodialysate of humans and rodents applied to pharmacokinetic studies in obese individuals	Morbidly obese patients (4)	2 g i.v. bolus	30 min after administration	n.a.	UF and HPLC-UV	M:68.3%	Observational study
Parker 2015	Determination of Cefalothin and Cefazolin in Human Plasma, Urine and Peritoneal Dialysate by UHPLC-MS/MS: application to a pilot pharmacokinetic study in humans	Patients with peritoneal dialysis-associated peritonitis (1)	15 mg/kg every 6 h by a 6h intraperitoneal dwell	1, 3, 6, 7 and 24 h post intraperitoneal antibiotic administration	n.a.	UF and UHPLC-MS/MS	R:79-82%	Pharmacokinetic study

	Roberts 2015	Plasma and target-site subcutaneous tissue population pharmacokinetics and dosing simulations of cefazolin in post-trauma critically ill patients	Critically ill adult patients (30)	1 g i.v. in 5 min	Pre-dose, at 5 min (end of infusion) and at 20, 60, 210 and 360 min	n.a.	UF and HPLC-MS	60% ± 12% at 5 min<br>73% ± 10% at 1 h<br>77% ± 9% at 6 h	Observational pharmacokinetic study
	Roberts 2016	Pharmacokinetics of Intraperitoneal Cefalothin and Cefazolin in Patients Being Treated for Peritoneal Dialysis-Associated Peritonitis	Patients with peritoneal dialysis-associated peritonitis (11)	15 mg/kg once daily in a 6 h intraperitoneal dwell	1, 3, 6, 7 and 24 h after the intraperitoneal administration	From an indwelling peripheral intravenous cannula	UF and UHPLC-MS/MS	M:63.9% (I:57.6-66.3%)	Prospective observational pharmacokinetic study
	Rowan 2017	Wound Penetration of Cefazolin, Ciprofloxacin, Piperacillin, Tazobactam, and Vancomycin During Negative Pressure Wound Therapy	Burn and trauma patients with negative pressure wound therapy (8)	Every 8 h	0, 0.5, 2, 4, and 8 h after administration	n.a.	UF and HPLC-UV	86.4% ± 6.9%	Prospective, observational study
	Van Kralingen 2011	Pharmacokinetics and protein binding of cefazolin in morbidly obese patients	Morbidly obese patients scheduled to undergo laparoscopic gastric banding or	2 g i.v. bolus	30, 180 and 240 min after cefazolin dosing and 2h after wound closure	n.a.	UF and HPLC-UV	M:79% (I:74-82%)	Prospective study

			gastric bypass surgery (20)					
	Vella-Brincat 2007	Protein binding of cefazolin is saturable in vivo both between and within patients	Patients treated with cefazolin i.v. by continuous infusion or intermittent injection (31)	Study i: 3 g 24 h-1<br><br>Study ii: intermittent 1 or 2 g i.v. bolus	Study i: any time in the 24 h infusion period. In patients with intermittent boluses samples were taken at the trough concentration<br><br>Study ii:<br>Trough: prior to a dose<br>Peak: 15-20 min after administration	n.a.	UF and HPLC-UV	Study I: 81.4% (R:49.4-90.2%)<br>Study ii: trough 81.3% and peak 68.7%<br>From low concentrations (8.5 mg l^{-1}): 91% to 49% at high concentrations (140 mg l^{-1})	Observational study
**Cefazolin in serum**	Brodwall 1977	Kidney transport of cefazolin in normal and impaired renal function	Patients with impaired renal function (9)	500 mg i.m.	n.a.	n.a.	ED and microbiological assay	86.9% ± 1.84	Observational study
	Craig 1973	Pharmacology of Cefazolin and Other Cephalosporins in Patients with Renal Insufficiency	Healthy volunteers (6) and patients with various degrees of renal impairment (11)	The six normal subjects and two patients with creatinine clearances of 15 ml/min and 10 ml/ min: multiple doses during	0, 0.5, 1, 2, 4, 6, 8, and 12 h after administration<br>From patients with significant renal impairment, additional samples were drawn at 24, 36, and 48 h after administration	Venous blood sampling	ED and microbiological assay	Healthy volunteers: 83.9% ± 1.4%<br>Patients: R:61.6-84.7%	Observational study

				a four-day period. Normal volunteers: 500 mg i.m. every 8 h. Uremic patients: 500 mg loading dose and 250 mg every 12 h or 24 h					
	De Cock 2017	Population pharmacokinetics of cefazolin before, during and after cardiopulmonary bypass to optimize dosing regimens for children undergoing cardiac surgery	Children undergoing cardiac surgery with CPB (56)	25 mg/kg i.v. as a bolus 1 h before surgical incision, at the start of rewarming on CPB, 8 h after the second dose and h after the third dose	Before, during and after CPB	Through an arterial catheter	UF and HPLC-UV	M:72% (I:64-77%)	Prospective pharmacokinetic study
	Himebauch 2014	Skeletal muscle and plasma concentrations of cefazolin during cardiac surgery in infants	Children undergoing CPB (12)	25 mg/kg i.v. within 1 h before incision and 25 mg/kg added to the CPB priming volume	Before and 5, 15, 30, 60, 90, and 120 min after administration, immediately before and after DHCA, and at sternum and skin closure	Through an existent arterial catheter of from the CPB circuit	UF and HPLC-MS	M:84.8% (I: 79.8-88.0%) Lower during DHCA: M:78.9% (I:77.3%-81.9%)	Pharmacokinetic study

Hites 2016	Evaluation of total body weight and body mass index cut-offs for increased cefazolin dose for surgical prophylaxis	Patients undergoing gastric bypass surgery, partial hepatectomy, duodenopancreatectomy or colectomy (63)	2 g i.v. over 30 min	Immediately prior to the administration, at 30, 60, 120 and 180 min after administration and a the end of the surgical procedure	Through a central venous line	UF and UPLC-UV	BMI < 35: 78.4%<br>BMI ≥ 35: 79.1%<br>TBW < 120 kg: 79.8%<br>TBW ≥120 kg: 79.6%	Prospective study
Hollis 2015	Validation of a Dosing Strategy for Cefazolin for Surgery Requiring Cardiopulmonary Bypass	Patients with CPB (10)	2 g within 1 h of incision, an additional 1 g injected into the bypass circuit at the onset of bypass, and 2 g every 3 h after the initial dose	Immediately after incision,<br>10 min after the start of bypass, each hour of bypass, at the end of bypass, and at sternal closure	From the bypass circuit when the patient was on bypass and from a central venous catheter when the patient was not on bypass	UF and HPLC	R:53-75%	Prospective, open label study
Koshida 1987	Comparative distribution kinetics of cefazolin and tobramycin in children	Children, age 3-12 y undergoing examination with cardiac catheterization (5)	25 mg/kg i.v. infusion over 20 min	30, 50, 70, 90, 110, 130, 190, 220 and 280 min after the start of the infusion	Through an indwelling needle placed in a vein of the dorsal manus	UF and HPLC-UV	78.1% ± 2.5%	Observational study
Ohashi 1986	Pharmacokinetics and protein binding of cefazolin and cefalothin in patients with cirrhosis	Patients with cirrhosis (12) or hepatitis (8) and normal	1 g i.v. single bolus	Before administration, and at 0.25, 0.5, 1, 2, 4 and 6 h after administration	Venous blood sampling	UF and microbiological assay	Cirrhotic patients: 72.3 ± 8.5%<br>Hepatitis patients: 84.7 ± 3.3%	Observational study

			volunteers (12)					Control group: 88.6 ± 1.9%	
**Cefradine dialysate**	Martea 1987	Pharmacokinetics of cefradine, sulfamethoxazole and trimethoprim and their metabolites in a patient with peritonitis undergoing continuous ambulatory peritoneal dialysis	54 y male with continuous ambulatory peritoneal dialysis for end-stage renal failure with peritonitis (1)	500 mg 4 times daily	n.a.	Venous blood sampling	UF and HPLC	6.1% ± 2.8%	Case report
**Cefradine pericardial fluid**	Nightingale 1980	Effect of Protein Binding on the Penetration of Nonmetabolized Cephalosporins into Atrial Appendage and Pericardial Fluids in Open-Heart Surgical Patients	Patients undergoing coronary artery bypass or cardiac valve replacement (17)	2 g i.v (30-118 min before the removal of the right atrial appendage	Immediately upon opening the pericardium	n.a.	UF and microbiological assay	< 10%	Observational study
**Cefradine plasma**	Martea 1987	Pharmacokinetics of cefradine, sulfamethoxazole and trimethoprim and their metabolites in a patient with peritonitis undergoing continuous	54 y male with continuous ambulatory peritoneal dialysis for end-stage renal failure with peritonitis (1)	500 mg 4 times daily	At fixed time intervals	Venous blood sampling	UF and HPLC	29.1% ± 6.6%	Case report

		ambulatory peritoneal dialysis							
**Cefalothin in pericardial fluid**	Green 1981	A Comparison of the Penetration Characteristics of Cephapirin and Cefalothin into the Right Atrial Appendage, Muscle, Fat, and Pericardial Fluid of Pediatric Patients Undergoing Open-Heart Operation	Pediatric patients (2-12 y) (32)	30 mg/kg i.v. injection 12 to 93 min prior to the removal of the right atrial appendage	Immediate after the opening of the pericardium	n.a.	UF and microbiological assay	79.6% ± 5.7% (< 1h after the administration in pericard eff.)	Observational study
**Cefalothin in plasma**	Parker 2015	Determination of Cefalothin and Cefazolin in Human Plasma, Urine and Peritoneal Dialysate by UHPLC-MS/MS: application to a pilot pharmacokinetic study in humans	Patients with peritoneal dialysis-associated peritonitis (1)	15 mg/kg by a 6 h intraperitoneal dwell	1, 3, 6, 7 and 24 h post intraperitoneal antibiotic administration	n.a.	UF and UHPLC-MS/MS	R:47-51%	Observational study
	Roberts 2016	Pharmacokinetics of Intraperitoneal Cefalothin and Cefazolin in Patients Being Treated for Peritoneal Dialysis-Associated Peritonitis	Patients with peritoneal dialysis-associated peritonitis (8)	15 mg/kg once daily in a 6 h intraperitoneal dwell	1, 3, 6, 7 and 24 h after the intraperitoneal administration	From an indwelling peripheral intravenous	UF and UHPLC-MS/MS	M:49% (I:45.1-55.1%)	Prospective observational pharmacokinetic study

**Cefalothin in serum**	Craig 1973	Pharmacology of Cefazolin and Other Cephalosporins in Patients with Renal Insufficiency	Healthy volunteers (6)	500 mg i.m.	0, 0.5, 1, 2, 4, 6, 8, and 12 h after the dose of antibiotic. From patients with significant renal impairment, additional samples were drawn at 24, 36, and 48 h after injection	Venous blood sampling	ED and microbiological assay	65.2% ± 4.3%	Observational study
	Ohashi 1986	Pharmacokinetics and protein binding of cefazolin and cefalothin in patients with cirrhosis	Patients with cirrhosis (12) or hepatitis (8) and normal volunteers (12)	1 g i.v. single bolus	Before, and at 0.25, 0.5, 1, 2, 4 and 6 h after administration	Venous blood sampling	UF and microbiological assay	Cirrhotic patients: 73.5 ± 4.01% Hepatitis patients: 75.0 ± 3.0% Control group: 75.9 ± 3.4%	Observational study
**Cefapirin in pericardial fluid**	Green 1981	A Comparison of the Penetration Characteristics of Cephapirin and Cefalothin into the Right Atrial Appendage, Muscle, Fat, and Pericardial Fluid of Pediatric Patients Undergoing Open-Heart Operation	Pediatric patients (2-17 y) (32)	30 mg/kg i.v. injection 12 to 93 min prior to the removal of the right atrial appendage	Immediate after the opening of the pericardium	n.a.	UF and microbiological assay	36.7% ± 10.7% (<1h after the administration in pericard eff.)	Observational study
**<u>Second generation cephalosporins</u>**									

**Cefamandole in plasma**	Berkhout 2003	Clinical Pharmacokinetics of Cefamandole and Ceftazidime Administered by Continuous Intravenous Infusion	Patients treated with cefamandole (10)	Continuous i.v. infusion 4 g daily	2 samples 1 h apart at least 6 h after initiation of treatment by continuous infusion After the second sample was taken, the dose was doubled, at least 6 h later, two blood samples were again taken 1 h apart	Venipuncture from the arm opposite to that used for the infusion of the antibiotic	ED and HPLC-UV	M:68% (R:62-75%)	Observational study
**Cefonicid in wound drainage**	Swanson 1991	Cefonicid versus Clindamycin Prophylaxis for Head and Neck Surgery in a Randomized, Double-Blind Trial, with Pharmacokinetic Implications	Patients undergoing oncologic head and neck surgery (6)	1 g 1-2 h single dose preoperatively	Intervals of 0 to 2, 2 to 4, 4 to 8, 8 to 12, and 12 to 24 h following wound closure	Continuous-drainage catheter in the deep compartment of the wound	Ultrafiltration and HPLC-UV	85%	Prospective, randomized, double-blind trial
**Cefonicid in serum**	Ackerman 1988	Disposition of cefonicid in orthopedic surgery patients	Patients undergoing hip reconstructive procedures (10) and controls (4)	15 mg/kg single dose	0.5, 4 and 12 h postinfusion	Venous blood sampling	UF and HPLC	Patients: 88% ± 5.41% after 0.5 h, 92.97% ± 6.56% after 4 h and 96.54% ± 1.44% after 12 h Controls: 93.02% ± 1%	Observational study

								after 0.5 h and 96.04% ± 1.79% after 4 h Overall: 92.0 ± 16%.	
	Dudley 1986	Effect of Saturable Serum Protein Binding on the Pharmacokinetics of Unbound Cefonicid in Humans	Healthy volunteers (6)	30 mg/kg single bolus i.v. infusion	0, 5, 10, 15, 20, 30, 45, 60, 75, and 90 min and 2, 2.5, 3, 3.5, 4, 5, 6, 8, 12, and 24 h after administration	Through an indwelling intravenous catheter during the first 8 to 12 h and then by direct venipuncture	UF and microbiological assay	82.4 ± 6.1% immediately after administration and 98% for total serum concentrations < 100 ug/ml.	Observational study
	Swanson 1991	Cefonicid versus Clindamycin Prophylaxis for Head and Neck Surgery in a Randomized, Double-Blind Trial, with Pharmacokinetic Implications	Patients undergoing oncologic head and neck surgery (59)	1 g 1-2 h single dose preoperatively	0, 0.5, 1, 1.5, 2, 4, 6, 8, 10, 12, 18, and 24 h after administration	n.a	UF and HPLC-UV	89%	Prospective, randomized, double-blind trial
	Trang 1989	Effect of Age and Renal Function on Cefonicid Pharmacokinetics	Geriatric hospitalized male subjects with urinary tract infection (10) and young non-hospitalized male subjects	15 mg/kg i.v. infusion of 15 min	0.5, 4, and 12 h	From the contralateral extremity	UF and HPLC-UV	Geriatric: 85.3 ± 8.5% after 0.5h, 96.4 ± 1.6% after 4h and 96.9 ± 1.6% after 12h Young: 93% ± 1% after	Observational study

			as controls (10)					0.5h, 96.9% ±0.3% after 4h	
**Ceforanide in serum**	DiPiro 1985	Intraoperative Ceforanide Pharmacokinetics and Protein Binding	Hospitalized adult patients scheduled for cholecystectomies (15)	20 mg/kg i.v.	At incision, 1h post incision and wound closure. For operations lasting longer than 2 h, additional intraoperative blood samples were collected hourly	n.a.	UF and HPLC-UV	87.9%	Observational study
**Cefotetan in plasma**	Yates 1983	Pharmacokinetics and tolerance of angle intravenous doses of cefotetan disodium in male Caucasian volunteers	Healthy male Caucasian volunteers (10)	2 g i.v. injection over 3 min	At 0, 10, 60 min and 3 and 9 h after administration	n.a.	ED and HPLC-UV/microbiological assay	88% (R:78-91%)	Observational study
**Cefotetan in serum**	Carver 1989	Pharmacokinetics and pharmacodynamics of total and unbound cefoxitin and cefotetan in healthy volunteers	Healthy volunteers (6)	2 g i.v. infusion over 5 min	Prior to administration and at times 0 (end of infusion) 5, 10, 15, 30, 45 min and 1, 1-5, 2, 3, 4, 6, 8, 12 and 24 h after administration	Venous blood sampling	UF and microbiological assay	85% ± 4.2%	Observational study
**Cefoxitin in blister fluid**	Wise 1980	The Influence of Protein Binding upon Tissue Fluid Levels of Six β-Lactam Antibiotics	Healthy volunteers	1 g i.m.	At 2 h after administration	Trough superficial cantharides-induced blisters which were emptied with use of a	UF and microbiological assay	59%	Observational study

						fine-bore needle and syringe			
**Cefoxitin in plasma**	Garcia 1979a	Pharmacokinetics of Cefoxitin in patients undergoing hemodialysis	Patients with terminal renal impairment and undergoing 6 h HD sessions (10)	15 mg/kg at the beginning of the 6 h HD sessions	0, 0.16, 0.5, 0.75, 1, 2, 3, 5, 6 h	n.a.	Method for separation bound/free not known and microbiological assay	41.46% (R:31.03-50%) (during HD)	Pharmacokinetic study
	Garcia 1979b	Pharmacokinetics of Cefoxitin in Patients with Normal or Impaired Renal Function	Patients with normal renal function (10) and varying degrees of renal impairment (13) and patients with terminal impairment (7)	15 mg/kg single i.v. injection	Normal: 0, 0.16, 0.5, 0.75, 1, 1.5, 2 and 3 h<br>Impaired and terminal: 0, 0.5, 1, 3, 6, 9, 12, 15 and 24 h	n.a.	UF and microbiological assay	73.22% normal renal function, decreasing with CrCl to 20-40% at CrCl of 20 ml/min	Observational study
**Cefoxitin in serum**	Carver 1989	Pharmacokinetics and pharmacodynamics of total and unbound cefoxitin and cefotetan in healthy volunteers	Healthy volunteers (6)	2 g i.v. infusion over 5 min	Prior to administration and at times 0 (end of infusion) 5, 10, 15, 30, 45 min and 1, 1-5, 2, 3, 4, 6, 8, 12 and 24 h after administration	Venous blood sampling	UF and microbiological assay	52% ± 2.8%	Observational study
**Cefuroxime in blister fluid**	Wise 1980	The Influence of Protein Binding upon Tissue Fluid Levels of	Healthy male volunteers (6)	1 g i.m.	At 2 h after administration	Trough superficial cantharides-induced	UF and microbiological assay	34%	Observational study

		Six β-Lactam Antibiotics				blisters which were emptied with use of a fine-bore needle and syringe			
**Cefuroxime in plasma**	Aalbers 2015	Targeting cefuroxime plasma concentrations during coronary artery bypass graft surgery with cardiopulmonary bypass	Patients undergoing CABG (21)	The first dose, 1500 mg of cefuroxime i.v., was given with anesthesia induction. The second dose, 750 mg i.v., was given 1 h after the first dose (t = 1 h). The dosing regimen was continued with cefuroxime 750 mg every hour until wound closure (t = 2; t = 3; t = 4; t = 5)	Trough samples were taken just before the next dose; peak samples were taken 30 min after the dose. Samples were also drawn at time of skin incision, at start of CPB, every 30 min of CPB, at the end of CPB and at wound closure	Blood samples were collected from the radial artery catheter or directly arterial from the CPB system	UF and HPLC	27.5% ± 5.0 %	Prospective study
	Mandak 2007	Tissue and plasma concentrations of cephuroxime during cardiac surgery in	Patients undergoing CABG (9)	3 g i.v. was given with anesthesia induction,	Before surgery, at the beginning of the CPB, every 30 min of the CPB, at	n.a.	UF and HPLC-UV-PDA	16.3%	Prospective study

	cardiopulmonary bypass--a microdialysis study		then 1.5 g i.v. after CPB , and 1.5 g i.v. 8 h after surgery	the end of the CPB and at the end of the surgery				
Van Raaij 2020	Quantification of total and unbound cefuroxime in plasma by ultra-performance liquid chromatography tandem mass spectrometry in a cohort of critically ill patients with hypoalbuminemia and renal failure	Critically ill patients with hypoalbuminemia and renal failure (11)	i.v. dosing regimens were prescribed by the attending physician	Trough: 180-1 min prior to administration<br>Peak: 10-30 min after administration<br>Mid: 30360 min after administration and sampling during continuous administration	n.a.	UF and UPLC-MS/MS	M:24.58% (R:0.25-72.64%)	Prospective, noninterventional feasibility study
Verhagen 1994	The renal clearance of cefuroxime and ceftazidime and the effect of probenecid on their tubular excretion	Healthy male volunteers (6)	First session: 750 mg i.v. followed by a continuous i.v. infusion of 420 mg/h<br>Second session: i.v. bolus of 375 mg followed by a continuous infusion of 210 mg h-1; after 2.5 h the	First session: before drug administration and every 30 min for 6.5 h<br>Second session: every 30 min for 7.5 h	Through an i.v. catheter	ED and HLPLC	17.2% ± 4.2%	Observational study

				bolus injection of 375 mg was repeated, followed by a continuous infusion of 420 mg h-'; after 5 h a bolus injection of 750 mg was given, followed by a continuous infusion of 840 mg h-1.					
**Cefuroxime in serum**	Foord 1976	Cefuroxime: Human Pharmacokinetics	Normal male subjects (5)	1 g i.m.	30, 60, 90, 120, 180, 240, 360, and 480 min	Venous blood sampling	UF and microbiological assay/HPLC	33% ± 5.7% No difference between young and elderly	Observational study
<u>**Third generation cephalosporins**</u>									
**Cefixime in serum**	Faulkner 1988	Pharmacokinetics of cefixime in the young and elderly	Young (12) and elderly subjects (12)	400 mg oral once-a-day for 5 days	Before and at 4 and 12 h after administration on days 1 and 5	Venipuncture	ED and HPLC-UV	67%	Observational study
**Cefmenoxime in serum**	Reitberg 1984	Effect of protein binding on cefmenoxime steady-state kinetics in critical patients	Critical patients with gram-negative pneumonia (20)	16 patients were given 1 g every 6 h, 2 received 2 g every 6 h, and	At steady stage over one dosing interval before administration, immediately after	Venous blood samples	ED and HPLC-UV	43.5% ± 13.0%	Observational study

				2 received 2 g every 8 h i.v. infusion over 5 min	administration, and 0.25, 0.5, 1, 2, 3, 4, 6, and 8 (where applicable) h after administration				
**Cefodizime in bronchial secretion**	Scaglione 1997a	Serum Protein Binding and Extravascular Diffusion of Cefodizime and Ceftriaxone An In Vivo Study	Patients with acute exacerbation of chronic bronchitis (13)	1g i.m. every day	On the second day of the treatment just before and at 2, 4, 8 and 12 h after administration	Velocity gradient technique	UF and HPLC-UV	68.1% at 2h<br>68.8% at 4h<br>70.2% at 8h<br>73.1% at 12h	Observational study
**Cefodizime in skin blister fluid**	Schäfer-Korting 1986	Cefodizime Penetration into Skin Suction Blister Fluid Following a Single Intravenous Dose	Healthy male volunteers (6)	1 g i.v. bolus	3, 6 and 9 h, in subject 1-3 also at 27 h	2 suction cups with altogether 10 holes (diameter 8 ram) were fixed to the abdominal wall at 0.5, 3.5 and 6.5 h, and a negative pressure of -250 mm Hg was raised slowly and maintained for 2.5 h	UF and microbiological assay	61.6% ± 2.7%	Observational study
**Cefodizime in serum**	Scaglione 1997a	Serum Protein Binding and Extravascular Diffusion of Cefodizime and	Patients with acute exacerbation of chronic	1g i.m. every day	On the second day of the treatment just before and at 2, 4, 8 and 12 h	n.a.	UF and HPLC-UV	81.1% at 2h<br>81.3% at 4h<br>82.9% at 8h<br>84% at 12h	Observational study

		Ceftriaxone An In Vivo Study	bronchitis (13)		after administration				
	Scaglione 1997b	Pharmacokinetic Study of Cefodizime and Ceftriaxone in Sera and Bones of Patients Undergoing Hip Arthroplasty	Patients undergoing hip arthroplasty (22)	2 g i.v. in 30 min before surgery	n.a.	From the surgical field	UF and HPLC-UV	85.45%	Observational study
	Schäfer-Korting 1986	Cefodizime Penetration into Skin Suction Blister Fluid Following a Single Intravenous Dose	Healthy male volunteers (6)	1 g i.v. bolus	Before drug administration and at 5, 15, 30 and 45 min and 1, 1.5, 2, 3, 4.5, 6, 7.5, 9, 24 (in Subjects 1-3 also 27) h after administration	From the cubital vein of the other arm	ED and microbiological assay	81%	Observational study
**Cefoperazone in plasma**	Kan 2020	An adapted LC-MS/MS method for the determination of free plasma concentration of cefoperazone in children: Age-dependent protein binding	Pediatric children: newborns (17), infants (10) and children (19)	Newborns: 78.9 (39.1–131.0) mg/kg/day Infants: 85.9 (41.7–233.4) mg/kg/day Children: 96 (46.5–100.0) mg/kg/day	Opportunistic sampling strategy	n.a.	UF and LC-MS/MS	Overall: 83.3% (R:52-91.9%) Newborn: 74.5% ± 9.1% Infants: 82.2% ± 7.1% Children: 87.5% ± 3.2% Lower albumin: 74.5% ± 9.7% Higher albumin: 84.4% ± 6.7%	Observational study
	Lam 1988	Effect of Protein Binding on Serum Bactericidal Activities	Healthy male volunteers (6)	30 mg/kg single dose in	0, 1, 2, 4, 8 and 12 h after the start of the infusion	n.a.	UF and HPLC-UV	91.5% ± 2.0%	Observational study

		of Ceftazidime and Cefoperazone in Healthy Volunteers		a 30 min infusion					
	Rao 2020	Determination of Total and Unbound Meropenem, Imipenem/Cilastatin, and Cefoperazone/Sulbactam in Human Plasma: Application for Therapeutic Drug Monitoring in Critically Ill Patients	Critically ill patients (8)	3 g every 8 h	At steady state defined as sampling after administration of at least 4 prior doses. For continuous infusion, plasma samples were obtained after at least 4 half-lives	n.a.	UF and LC-MS/MS	R:79.74-99.14%	Validation study / observational
**Cefoperazone in serum**	Kalman 1992	Pharmacokinetic Disposition and Bactericidal Activities of Cefepime, Ceftazidime, and Cefoperazone in Serum and Blister Fluid	Healthy male volunteers (6)	2 g i.v. single dose over 30 min	At the end of 0.5 h infusion and 2, 4, 8, 12 h after the start of infusion	n.a.	UC and HPLC-UV	Ca 90%	Observational study
**Cefotaxime in pleural exudate**	Scaglione 1990	Serum protein binding and extravascular diffusion of methoxyimino cephalosporins. Time courses of free and total concentrations of cefotaxime and ceftriaxone in serum and pleural exudate	Patients with pleural empyema treated by intercostal drainage (12)	1 g i.v. bolus	0.5, 1, 1.5, 2, 3, 4 and 6 h after dosing	Intercostal drain	ED and microbiological assay	7.63%	Observational study

**Cefotaxime in peritoneal fluid**	Seguin 2009	Plasma and peritoneal concentration following continuous infusion of cefotaxime in patients with secondary peritonitis	Critically ill patients with secondary peritonitis (11)	Continuous infusion of 4 g/24h after a loading dose bolus of 2 g	On day 3	Exteriorized by surgical drainage (vacuum suction system using a redon catheter or latex drainage tube)	UF and HPLC-UV	12.9% on day 3	Prospective observational study
**Cefotaxime in plasma**	Dahyot-Fizelier 2013	Microdialysis Study of Cefotaxime Cerebral Distribution in Patients with Acute Brain Injury	Patients with acute brain injury (5)	2 g 3 times per day	Between 0.25 h and 0.75 h after the beginning of infusion and between 6 and 10 h	n.a.	UF and HPLC-UV	40.6% (R:32-52.6%)	Observational study
	Seguin 2009	Plasma and peritoneal concentration following continuous infusion of cefotaxime in patients with secondary peritonitis	Critically ill patients with secondary peritonitis (11)	Continuous infusion of 4 g/24h following a loading dose bolus of 2 g	At steady state on days 2 and 3	n.a.	UF and HPLC-UV	18.2%±5.9% on day 2 and 17.4%±7.7% on day 3	Prospective observational study
**Cefotaxime in serum**	Aardema 2020	Continuous versus intermittent infusion of cefotaxime in critically ill patients: a randomized controlled trial comparing plasma concentrations	Critically ill patients (59)	Intermittent: 4 g every 6 h (29 patients) Continuous: 4 g/24h after a loading dose of 1 g (30 patients)	Intermittent: day 1 at 0 min, directly after infusion at 40 min, 1, 2, 4, 8, 12 and 24 h after start of administration on. After that, trough and peak levels were obtained once daily	From an indwelling arterial catheter	UF and LC-MS/MS	Intermittent: M:29.45% (I:25-34.78%) Continuous: M:29.86% (I:25.83-33.71%)	Randomized controlled single-centre study

					just before and 40 min after bolus infusion, respectively, until the end of treatment<br><br>Continuous: day 1 at 0 min, then at 40 min from start of infusion of the loading dose, i.e. immediately after completion of the loading dose. Subsequent samples were drawn at 1, 2, 4, 8, 12 and 24 h after start of administration on day 1. During the subsequent days of continuous infusion, samples were drawn every 12h, until the end of treatment on day 4				
	Scaglione 1990	Serum protein binding and extravascular diffusion of methoxyimino	Patients with pleural empyema treated by	1 g i.v. bolus	0.5, 1, 1.5, 2, 3, 4 and 6 h after dosing	Venous blood sampling from the antecubital fossa	ED and microbiological assay	9.93%	Observational study

		cephalosporins. Time courses of free and total concentrations of cefotaxime and ceftriaxone in serum and pleural exudate	intercostal drainage (12)						
**Cefpiramide in plasma**	Demotes-Mainard 1991	Pharmacokinetics and protein binding of cefpiramide in patients with alcoholic cirrhosis	Patients with cirrhosis and ascites (11) and healthy male volunteers (11)	1 g i.v. single dose	Patients: 0, 1, 5, 10, 15, 20, 30, and 45 min and 1, 1.5, 2, 3, 4, 5, 6, 8, 10, 12, 24, 36, 48, 60, and 72 h after administration Volunteers: before and 1, 5, 10, 15, 20, 30, and 45 min and 1, 1.5, 2, 3, 4, 5, 6, 8, 10, 12, 15, 18, 24, and 36 h after start h administration	Venous blood sampling	UF and HPLC	Patients: 89.6% ± 9.5% Volunteers: 98.1% ± 0.3%	Observational study
	Demotes-Mainard 1994	Cefpiramide kinetics and plasma protein binding in cholestasis	Patients with cholestasis (8)	1 g i.v. single dose	Before dosing and at 5, 10, 15, 20, 30, and 45 min and 1, 1.5, 2, 3, 4, 5, 6, 8, 10, 12, 15, 18, 24 and 36 h after administration	Venous blood sampling	UF and HPLC-UV	77% ± 0.13%	Observational study
**Cefpiramide in serum**	Conte 1987	Pharmacokinetics of Cefpiramide in Volunteers with Normal or Impaired Renal Function	Healthy volunteers (10), patients with normal or renal impairment	2 g i.v.	1, 2, 4, and 8 h after start of the infusion	n.a.	UF and HPLC	Volunteers: 92.2% ± 1.4% - 99.3% ± 0.8% Patients with normal or	Observational study

			(10) and patients with chronic HD (10)					renal impairment: 91.1% ± 1.8% - 98.2% ± 0.8%<br>Patients with chronic HD: 88.5% ± 7.1% - 94.9% ± 4.8%	
**Ceftazidime in plasma**	Berkhout 2003	Clinical Pharmacokinetics of Cefamandole and Ceftazidime Administered by Continuous Intravenous Infusion	Patients treated with ceftazidime (5)	Continuous infusion i.v. 1.5 g daily but 3 g in case of infection with *Pseudomonas aeruginosa* was suspected	2 samples 1 h apart at least 6 h after initiation of treatment by continuous infusion<br>After the second sample was taken, the dose was doubled, at least 6 h later, two blood samples were again taken 1 h apart	Venipuncture from the arm opposite to that used for the infusion of the antibiotic	ED and HPLC-UV	0%	Observational study
	Isla 2007	In vitro AN69 and Polysulphone Membrane Permeability to Ceftazidime and in vivo Pharmacokinetics during Continuous Renal	Male patients in the ICU undergoing CVVH or CVVHD (4)	2 patients 1 g every 6 h<br>2 patients 2 g every 6 h infusion over 20 min	Immediately before dosing, at the end of the infusion and at 30 and 45 min and 1, 3, and 6 h after the infusion start	n.a.	UF and HPLC-UV	14% ± 8%	Observational study

		Replacement Therapies							
	Lam 1988	Effect of Protein Binding on Serum Bactericidal Activities of Ceftazidime and Cefoperazone in Healthy Volunteers	Healthy male volunteers (6)	30 mg/kg single dose in a 30 min infusion	0, 1, 2, 4, 8 and 12 h after the start of the infusion	Venous blood sampling	UF and HPLC-UV	21.0% ± 6.0%	Observational study
	Matzke 2000b	Determinants of Ceftazidime Clearance by Continuous Venovenous Hemofiltration and Continuous Venovenous Hemodialysis	Patients with end-stage renal disease receiving conventional maintenance HD (8)	1 mg i.v. single dose over 30 min infusion	n.a.	n.a.	UF and HPLC-UV	17% ± 6% (R:10-25%)	Observational study
	Schieser 2021	Measurement of Free Plasma Concentrations of Beta-Lactam Antibiotics: An Applicability Study in Intensive Care Unit Patients	ICU patients (7)	6g/24 h continuous infusion<br>1 patient 4 g/24h	2 at steady state: if the time interval between the start of the therapy was >16 h (if a loading dose was administered) or 32 h if no loading dose was administered, and at least 8 h after the end of renal replacement therapy	Arterial blood samples	UF and HPLC-UV	0%	Prospective observational study
**Ceftazidime in serum**	Van Dalen 1986	Dosage Adjustment for Ceftazidime in Patients with	Patients in an ICU with varying	1 g i.v. bolus injection	After 5, 10, 15, 30 and 45 min, and 1, 1.5, 2, 2.5, and 3,	From an indwelling radial or	UF and HPLC-UV	<8% except in 4 patients in whom it	Observational study

		Impaired Renal Function	degrees of renal function, including patients on regular HD (20)		then hourly until 10 h after administration, and thereafter once every 2 h up to 24 h after administration<br><br>Every fourth sample was measured for protein binding	femoral artery catheter		was 20-30% (R:0-31%)	
**Ceftizoxime in cord serum**	Fortunato 1993	Differential protein binding of ceftizoxime in cord versus maternal serum	25 samples of cord serum	n.a.	Cord blood at time of delivery	n.a.	UF and HPLC-UV	21.9% ± 0.04%	Observational study
**Ceftizoxime in maternal serum**	Fortunato 1993	Differential protein binding of ceftizoxime in cord versus maternal serum	25 samples of pregnant women	Three or more doses	With the first routine blood collection after the third dose	n.a.	UF and HPLC-UV	57.8% ± 0.04%	Observational study
**Ceftriaxone in cerebrospinal fluid**	Hoshino 2010	Free concentration and protein-binding ratio of ceftriaxone in cerebrospinal fluid in paediatric patients with purulent meningitis caused by Haemophilus influenzae type b	Pediatric patients with meningitis (2 months-5y) (12)	50 mg/kg i.v. in 30 min every 12 h	n.a because of the difficulty of sampling	n.a.	UF and microbiological assay	18.8% ± 6.21%	Observational study
**Ceftriaxone in**	Scaglione 1997a	Serum Protein Binding and Extravascular	Patients with acute exacerbation	1g i.m. every day	On the second day of the treatment just before and at	Velocity gradient technique	UF and HPLC-UV	80% at 2h<br>78.4% at 4h<br>79.7% at 8h	Observational study

**bronchial secretion**		Diffusion of Cefodizime and Ceftriaxone An In Vivo Study	of chronic bronchitis (12)		2, 4, 8, 12 and 24 h after administration			80.8% at 12h	
**Ceftriaxone in pleural exudate**	Scaglione 1990	Serum protein binding and extravascular diffusion of methoxyimino cephalosporins. Time courses of free and total concentrations of cefotaxime and ceftriaxone in serum and pleural exudate	Patients with pleural empyema treated by intercostal drainage (12)	1 g i.v. bolus	0.5, 1, 1.5, 2, 3, 4 and 6 h after dosing	Intercostal drain	ED and microbiological assay	60.56%	Observational study
**Ceftriaxone in plasma**	Bos 2018	Pharmacokinetics and pharmacodynamic target attainment of ceftriaxone in adult severely ill sub-Saharan African patients: A population pharmacokinetic modelling study	Severely ill adults in sub-Saharan African patients (88)	1 g i.v. via a venous catheter	Pre-dose, 30-120 min after administration and two random timepoints during dosing intervals	n.a.	UF and HPLC-MS	M:81% (I:71-87%)	Prospective, observational population pharmacokinetic study
	Ebisch 2020	Ceftriaxone Dosing in a Critically Ill Patient With Hypoalbuminemia During Continuous Venous Hemofiltration: Emphasis	Critically ill patient requiring CVVH (1)	2 g once daily	Over 40 h at 8 different time points	n.a.	UF and UPLC-MS/MS	M:32.3% (I:26.3-41.1%)	Case report

		on Unbound Pharmacokinetics							
	Gijsen 2021	Pharmacokinetic/Pharmacodynamic Target Attainment Based on Measured versus Predicted Unbound Ceftriaxone Concentrations in Critically Ill Patients with Pneumonia: An Observational Cohort Study	Critically ill patients with severe community-acquired pneumonia (31)	2 g every once daily as infusion over 30 min	Trough: within 60 min before the next infusion<br>Peak: 30 min after the end of the infusion	n.a.	ED and UHPLC-MS/MS	M:83% (R:50-94.7%)<br>At trough M:87% (R:78-94.7%)	Prospective observational cohort study
	Gregoire 2019	High-Dose Ceftriaxone for Bacterial Meningitis and Optimization of Administration Scheme Based on Nomogram	Patients with suspected bacterial meningitis (153)	70-100 mg/kg/day<br><br>Mean daily dose was 7 g = 95.7 mg/kg	n.a.	Venipuncture through a catheter	UF and HPLC-UV	M:92.43% (R:50.7-98.39%)	Population PK study from an open-label, prospective, multicenter study
	Hartman 2021	Current Ceftriaxone Dose Recommendations are Adequate for Most Critically Ill Children: Results of a Population Pharmacokinetic Modeling and Simulation Study	Critically ill children 0.1-16.7 years, median 2.5 (43)	100 mg/kg once daily infused over 30 min	Opportunistic blood sampling<br><br>Unbound ceftriaxone concentrations were measured in one selected sample per patient closest to the end of the dosing interval	n.a.	UF and HPLC-MS	M:86.4% (R:29.7-92.4%)	Two-center prospective population pharmacokinetic study

Herrera 2020	Is Once-Daily High-Dose Ceftriaxone plus Ampicillin an Alternative for *Enterococcus faecalis* Infective Endocarditis in Outpatient Parenteral Antibiotic Therapy Programs?	Healthy volunteers (12)	A. 2 g each 12 h over 30 min during 24 h<br><br>B. 4 g single dose over 30 min	Just before administration<br>A: 0.5, 1, 2, 3, 6, 8, 10, 12,<br>12.5, 13, 14, 15, 18, 20, 22, and 24 h after administration<br><br>B: 0.5, 1, 2, 3, 6, 8, 10, 12, 16, 20, and 24 h after administration	n.a.	UF and LC-MS/MS	A 93.38 ± 2.15<br>B 91.40 ± 5.65	Phase II, open-label, nonrandomized, crossover, pharmacokinetic study
Kan 2021	Prediction of Unbound Ceftriaxone Concentration in Children: Simple Bioanalysis Method and Basic Mathematical Equation	Children (92)	20-80 mg/kg once or twice a day	During routine biochemical tests	n.a.	UF and HPLC-UV	88.1% ± 6.3% (R:60-95.2%)	Observational study
Luderer 1983	Age and ceftriaxone kinetics	Young (8) and elderly (8)	1 g i.v. single dose infusion over 30 min	0, 10, 20, 30 (end of infusion), 35, 40, and 50 min and 1, 2, 4, 6, 8, 10, 12, 16, 24 and 48 h after starting the infusion	Venous blood sampling	ED and HPLC-UV	Young: 83% ± 2.4% at 0.5 h sample and 88.6% ± 1.2% at 4 h sample<br>Old: 77.8% ± 7.0% at 0.5 h sample and 85.4% ± 3.5% at 4h sample	Observational study

	Matzke 2000a	Determinants of Ceftriaxone Clearance by Continuous Venovenous Hemofiltration and Hemodialysis	Patients receiving HD (8)	1 g i.v. as 1 h infusion	At midpoint of each dialysate ultrafiltrate collection period	n.a.	UF and HPLC-UV	43 ± 15% (R:13–92%)	Prospective outpatient study
	Mimoz 2000	Ceftriaxone pharmacokinetics during iatrogenic hydroxyethyl starch-induced hypoalbuminemia: A model to explore the effects of decreased protein binding capacity on highly bound drugs	Patients with hydroxyethyl starch-induced hypoalbuminemia (11) and matched healthy volunteers as controls (11)	2 g over 15 min infusion	15 min after administration	Venous blood samples were drawn from the arm	UF and HPLC	Patients: M:82% (R:75-86%) Volunteers: M:90% (R:74-94%)	Observational study
	Neves 2018	Therapeutic Doses of Eltrombopag do not Inhibit Hepatic BCRP in Healthy Volunteers: Intravenous Ceftriaxone as a Model	Healthy adult volunteers (12)	1 g intravascular over 3 min	0, 0.5, 1, 2, 4, 8, 12, 18, 24, 32, 40 and 48 h after the start of the infusion	n.a.	Method for separation bound/free not known and LC-MS/MS	Eltrombopag 0 mg: 91.1% (86.3-94%) Eltrombopag 25 mg: 89.1% (86.8-91.3%)<br><br>Eltrombopag 50 mg: 90% (87-92.9%)	Observational study
	Popick 1987	Plasma protein binding of ceftriaxone	Healthy subjects (12)	2 g i.v. infusion over 30 min	1 h after administration	n.a.	ED and HPLC-UV	R:82.2-89.0%	Observational study

Schleibinger 2015	Protein binding characteristics and pharmacokinetics of ceftriaxone in intensive care unit patients	ICU patients (17)	2g i.v. infusion in 30 min	Opportunistically with the aim of capturing one or two dosing intervals, with three to four samples	n.a.	UF and HPLC-UV	M:67% (I:54.5-79.8%)	Observational study
Stoeckel 1984	Single-dose ceftriaxone kinetics in liver insufficiency	Normal subjects (8) and subjects with various degrees of chronic liver damage (alcoholic fatty liver, cirrhosis without ascites and cirrhosis with ascites (15)	1 g i.v. bolus	At frequent intervals up to 48 h after injection	From a vein of the contralateral arm by means of an indwelling intravenous cannula	ED and HPLC	Normal: 95% ± 0.8%<br>Fatty liver: 92.9% ± 2.1%<br>Cirrhosis without ascites: 90.9% ± 2.0%<br>Cirrhosis with ascites: 83.9% ± 6.1%	Observational study
Toth 1991	Pharmacokinetics of Ceftriaxone in Liver-Transplant Recipients	Adult orthotopic liver transplant patients (7)	2 g i.v. infusion over 15 min, 3-5 days after the liver transplant	7, 15 and 30 min and 1, 2, 3, 4, 6, 8, 10, 12, 18 and 24 h after the start of the infusion<br><br>5 plasma samples from each patient, representing approximately every other time point, were used to	n.a.	ED and HPLC-UV	R:44-95%	Observational study

				determine plasma protein binding				
Tsai 2016	Total and unbound ceftriaxone pharmacokinetics in critically ill Australian Indigenous patients with severe sepsis	Critically ill Australian Indigenous patients with severe sepsis (5)	1 g i.v. infusion over 30 min via a central venous catheter every 12 h	30, 90, 180, 360 and 720 min after the start of the infusion	From the existing arterial line	UF and UHPLC-MS/MS	R:57-86%	Prospective, observational pharmacokinetic study
Ulldemolins 2021	Once-daily 1 g ceftriaxone optimizes exposure in patients with septic shock and hypoalbuminemia receiving continuous veno-venous hemodiafiltration	Patients with septic shock and hypoalbuminemia receiving CVVH (8)	1000 mg q12h (n = 5), 2000 mg q24h (n = 2), and 2000 mg q12h (n = 1) as a 30 min bolus	After at least 24 h of CVVHDF and ceftriaxone therapy at -10 min, 0 min, 15 min, 90 min and between 4 and 8 h after the end of the infusion and just before the next dose	n.a.	UF and LC-MS/MS	56%	Prospective pharmacokinetic study
Wong 2018	Therapeutic drug monitoring of β-lactam antibiotics in the critically ill: direct measurement of unbound drug concentrations to achieve appropriate drug exposures	Critically ill patients (n.a.)	Empirical dosing regimen	At steady-state defined as sampling after administration of at least four prior doses<br>Intermittent: at mid-point of the dosing interval and immediately prior to re-dosing<br>Continuous: after at least four half-lives	n.a.	UF and HPLC-UV	R:83-95%	Prospective observational study

**Ceftriaxone in serum**	Bourget 1993	Pharmacokinetics and protein binding of ceftriaxone during pregnancy	Pregnant women with chorioamnionitis or pyelonephritis (9)	2 g every 24 h every 24 h	0, 0.5, 1, 2, 4, 8, 12, 16, 20, and 24 h after administration	Venous blood samples via a Cathlon catheter	UF and HPLC-UV	92.58 ± 14.2%	Observational study
	Fukumoto 2009	Pharmacokinetics of Ceftriaxone, a Third-Generation Cephalosporin, in Pediatric Patients	Pediatric patients with pneumonia (8)	50 mg/kg/d	1 h after the 1st drug infusion and just before the 2nd infusion	n.a.	UF and HPLC-UV	~80-90% for mid-concentrations	Observational study
	Heinemeyer 1990	Clearance of ceftriaxone in critical care patients with acute renal failure	Surgical intensive care patients with a bacterial infection of the bronchial tract with normal renal function (6) or with acute renal failure (5)	2 g a day by a 15 min infusion for a period of 7 days	Prior to the second administration and the last administration (day 2 and 7)	n.a.	ED and HPLC	Normal renal function: R:85.5-91.5% Acute renal failure: R:70.9-83.6%	Observational study
	Hoshino 2010	Free concentration and protein-binding ratio of ceftriaxone in cerebrospinal fluid in paediatric patients with purulent meningitis caused by Haemophilus influenzae type b	Pediatric patients with meningitis (2 months-5y) (7)	50 mg/kg i.v. in 30 min every 12 h	n.a because of the difficulty of sampling	n.a.	UF and microbiological assay	81.8% ± 8.35%	Observational study

	Meenks 2021	Liquid Chromatography-Tandem Mass Spectrometry to Monitor Unbound and Total Ceftriaxone in Serum of Critically Ill Patients	ICU patients with sepsis (5)	4 patients 2 g once daily and 1 patient 2 g twice daily	Median 16 h after start of the infusion	n.a.	UF and UPLC-MS/MC	M:70.9% (I:47.8-84.8%)	Observational, single centre study
	Scaglione 1997a	Serum Protein Binding and Extravascular Diffusion of Cefodizime and Ceftriaxone An In Vivo Study	Patients with acute exacerbation of chronic bronchitis (12)	1g i.m. every day	On the second day of the 1g/day treatment just before and at 2, 4, 8, 12 and 24 h after drug administration	n.a.	UF and HPLC-UV	92.5% at 2h<br>93% at 4h<br>93.6% at 8h<br>94% at 12h<br>93.8% at 24h	Observational study
	Scaglione 1997b	Pharmacokinetic Study of Cefodizime and Ceftriaxone in Sera and Bones of Patients Undergoing Hip Arthroplasty	Patients undergoing hip arthroplasty (20)	2 g intravenous in 30 min before surgery	n.a.	From the surgical field	UF and HPLC-UV	79.16%	Observational study
	Scaglione 1990	Serum protein binding and extravascular diffusion of methoxyimino cephalosporins. Time courses of free and total concentrations of cefotaxime and ceftriaxone in serum and pleural exudate	Patients with pleural empyema treated by intercostal drainage (12)	1 g i.v. bolus	0.5, 1, 1.5, 2, 3, 4 and 6 h after dosing	Venous blood sampling from the antecubital fossa	ED and microbiological assay	70.17%	Observational study

**Cefepime in plasma**	Al-Shaer 2020	Meropenem, Cefepime, and Piperacillin Protein Binding in Patient Samples	Patients in University of Florida Health Shands Hospital where the physicians requested drug concentrations to allow the optimization of treatment (36)	n.a.	n.a.	n.a.	UF and LC-MS	M:39% (R:29-61%)	Observational study
	Isla 2005	Cefepime and Continuous Renal Replacement Therapy (CRRT): In Vitro Permeability of Two CRRT Membranes and Pharmacokinetics in Four Critically Ill Patients	Male patients undergoing CVVH or CVVHD (2)	2 g i.v. every 8 h	At steady state Immediately before dosing, at the end of the infusion and at 30 and 45 min and 1, 3, 6 and 8 h after the start of the infusion	n.a.	UF and HPLC-UV	21% ± 9%	Observational study
**<u>Fourth generation cephalosporins</u>**									
**Cefepime in serum**	Kalman 1992	Pharmacokinetic Disposition and Bactericidal Activities of Cefepime, Ceftazidime, and Cefoperazone in Serum and Blister Fluid	Healthy male volunteers (6)	2 g i.v. single dose over 30 min	At the end of 0.5 h infusion and 2, 4, 8, 12 h after the start of infusion	n.a.	UC and HPLC-UV	Ca 20%	Observational study

**Cefluprenam (E1077) in plasma**	Nakashima 1994	Phase I study of E1077, a novel parenteral cephem antibiotic	Healthy male volunteers (36)	2000 mg i.v. infusion in 60 min	Before and 1, 2, 4 and 8 h after the beginning of the infusion	n.a.	UF and microbiological assay/HPLC-UV	14.5% ± 2.9%	Phase I study
<u>**Fifth generation cephalosporins**</u>									
**Ceftobiprole in plasma**	Barbour 2009	Soft-Tissue Penetration of Ceftobiprole in Healthy Volunteers Determined by In Vivo Microdialysis	Healthy volunteers (15)	500 mg i.v. infusion	After 2 h and 12 h	n.a.	UF and HPLC-MS/MS	21.7% ± 6.6%	Observational study
**Ceftolozane in plasma**	Kratzer 2019	Determination of total and free ceftolozane and tazobactam in human plasma and interstitial fluid by HPLC-UV	Healthy volunteers (n.a.)	1 g i.v. infusion over 1 h	n.a.	n.a.	UF and HPLC-UV	6.3% ± 2.0%	Validation study, application to a pharmacokinetic study

Abbreviations: CABG = coronary artery bypass graft, CPB = cardiopulmonary bypass, CVVD = continuous venovenous hemodialysis, CVVH = continuous venovenous hemofiltration, DHCA = deep hypothermic circulatory arrest, ED = equilibrium dialysis, ECMO = extracorporeal membrane oxygenation, h = hour, HD = hemodialysis , HPLC = high-performance liquid chromatography, HPLC-MS = high-performance liquid chromatography-mass spectrometry, HPLC-MS/MS = high-performance liquid chromatography with tandem mass spectrometry, HPLC-UV = high-performance liquid chromatography with ultraviolet detection, HPLC-UV-PDA = high-performance liquid chromatography with ultraviolet photodiode-array detection, ICU = intensive care unit, I = interquartile range, i.m. = intramuscular i.v. = intravenous, LC-MS/MS = liquid chromatography with tandem mass spectrometry, M = median, n.a. = not available, NICU = neonatal intensive care unit, R = range, UC = ultracentrifugation, UF = ultrafiltration, UHPLC-MS/MS = ultra-high-performance liquid chromatography with tandem mass spectrometry
