# Supplementary material for: An Overview of the Protein Binding of Cephalosporins in Human Body Fluids: A Systematic Review
Source: Front Pharmacol. 2022 Jun 28;13:900551. doi: 10.3389/fphar.2022.900551 (PMC9274189; doi:10.3389/fphar.2022.900551)
Supplement: Supplementary file 2 [file DataSheet1.docx]

**Search strategy and datasources, n = number of records retrieved**

**Embase.com n=1565**

('beta lactam'/exp OR 'beta lactam antibiotic'/exp OR (beta-lactam OR beta-lactams OR β-lactam OR β-lactams OR biapenem* OR brobactam* OR carbapenem* OR clavulanate* OR clavulanic-acid* OR doripenem* OR durlobactam* OR ertapenem* OR fropenem* OR imipenem* OR l-786392* OR lenapenem* OR meropenem* OR monobactam* OR nacubactam* OR nocardicin-A* OR nocardicin-E* OR nocardicinic-acid* OR panipenem* OR penicillin* OR pirazmonam* OR razupenem* OR ritipenem* OR sanfetrinem* OR sulbactam* OR sulopenem* OR sultamicillin* OR taniborbactam* OR tazobactam* OR tebipenem* OR thienamycin* OR timentin* OR tomopenem* OR tribactam* OR trinem* OR aztreonam* OR adicillin* OR almecillin* OR aminopenicillin* OR amoxicillin* OR ampicillin* OR ancremonam* OR apalcillin* OR aspoxicillin* OR azidocillin* OR azlocillin* OR bacampicillin* OR bacmecillinam* OR carbacephem* OR carbenicillin* OR carfecillin* OR carindacillin* OR carumonam* OR cefacetrile* OR cefaclor* OR cefadroxil* OR cefalexin* OR cefaloglycin* OR cefaloram* OR cefaloridine* OR cefalotin* OR cefamandole* OR cefapirin* OR cefatrizine* OR cefazaflur* OR cefazedone* OR cefazolin* OR cefbuperazone* OR cefcanel* OR cefcapene* OR cefclidin* OR cefdaloxime* OR cefdinir* OR cefditoren* OR cefepime* OR cefetamet* OR cefetecol* OR cefiderocol* OR cefixime* OR cefluprenam* OR cefmatilen* OR cefmenoxime* OR cefmetazole* OR cefminox* OR cefodizime* OR cefonicid* OR cefoperazone* OR ceforanide* OR cefoselis* OR cefotaxime* OR cefotetan* OR cefotiam* OR cefovecin* OR cefoxitin* OR cefozopran* OR cefpimizole* OR cefpiramide* OR cefpirome* OR cefpodoxime* OR cefprozil* OR cefquinome* OR cefradine* OR cefroxadine* OR cefsulodin* OR ceftaroline* OR ceftazidime* OR cefteram* OR ceftezole* OR ceftibuten* OR ceftiofur* OR ceftizoxime* OR ceftobiprole* OR ceftolozane* OR ceftriaxone* OR cefuroxime* OR cefuzonam* OR cephalosporin* OR cephamycin* OR cloxacillin* OR cyclacillin* OR deacetoxycephalosporin* OR deacetylcefotaxime* OR deacetylcephalosporin* OR dicloxacillin* OR enmetazobactam* OR epicillin* OR fleroxacin-deacetylcefotaxime-ester* OR flomoxef* OR flucloxacillin* OR flumoxil* OR fomidacillin* OR furbenicillin* OR fuzlocillin* OR gloximonam* OR hetacillin* OR isopenicillin* OR latamoxef* OR lenampicillin* OR loracarbef* OR mecillinam* OR metampicillin* OR meticillin* OR mezlocillin* OR miraxid* OR nafcillin* OR nitrocefin* OR optocillin* OR oxacillin* OR penamecillin* OR penethamate* OR penicillic-acid* OR penicillin* OR penicilloic-acid* OR pheneticillin* OR piperacillin* OR pivampicillin* OR pivmecillinam* OR propicillin* OR quinacillin* OR retacillin* OR sulbenicillin* OR sulfazecin* OR talampicillin* OR tameticillin* OR temocillin* OR thiophenoxycefalotin* OR ticarcillin* OR tigemonam* OR tobicillin* OR triplopen* OR ureidopenicillin*):ab,ti) AND ('protein binding'/exp OR 'drug protein binding'/de OR ((binding NEAR/6 protein) OR Free-fraction* OR bound-fraction* OR unbound-fraction*):ab,ti) AND ('body fluid'/exp OR 'cerebrospinal fluid analysis'/de OR 'drug cerebrospinal fluid level'/exp OR 'drug blood level'/exp OR 'drug lymph level'/de OR 'drug peritoneal fluid level'/exp OR 'drug urine level'/exp OR 'blood analysis'/exp OR urinalysis/exp OR 'protein analysis'/de OR 'blood level'/de OR 'protein blood level'/de OR (((body OR ascites* OR blister* OR cerebrospinal* OR dentinal* OR duodenum* OR edema* OR epithelial-lining* OR extracellular* OR extravascular* OR follicle* OR intestine* OR intracellular* OR lung-extravascular* OR lung* OR ovary-follicle* OR peritoneal* OR pleura* OR synovial* OR transcellular* OR uterus* OR wound* OR interstitial*) NEAR/3 fluid*) OR blood* OR plasma OR serum* OR endolymph* OR perilymph* OR lymph* OR hemolymph* OR urine* OR aqueous-humor*):ab,ti) NOT ('antibiotic resistance'/exp/mj OR (resistan*):ti) NOT (([animals]/lim) NOT [humans]/lim) NOT ('in vitro study'/exp OR in-vitro:ti) NOT [conference abstract]/lim

**Medline ALL Ovid n=156**

(exp beta-Lactams/ OR (beta-lactam OR beta-lactams OR biapenem* OR brobactam* OR carbapenem* OR clavulanate* OR clavulanic-acid* OR doripenem* OR durlobactam* OR ertapenem* OR fropenem* OR imipenem* OR l-786392* OR lenapenem* OR meropenem* OR monobactam* OR nacubactam* OR nocardicin-A* OR nocardicin-E* OR nocardicinic-acid* OR panipenem* OR penicillin* OR pirazmonam* OR razupenem* OR ritipenem* OR sanfetrinem* OR sulbactam* OR sulopenem* OR sultamicillin* OR taniborbactam* OR tazobactam* OR tebipenem* OR thienamycin* OR timentin* OR tomopenem* OR tribactam* OR trinem* OR aztreonam* OR adicillin* OR almecillin* OR aminopenicillin* OR amoxicillin* OR ampicillin* OR ancremonam* OR apalcillin* OR aspoxicillin* OR azidocillin* OR azlocillin* OR bacampicillin* OR bacmecillinam* OR carbacephem* OR carbenicillin* OR carfecillin* OR carindacillin* OR carumonam* OR cefacetrile* OR cefaclor* OR cefadroxil* OR cefalexin* OR cefaloglycin* OR cefaloram* OR cefaloridine* OR cefalotin* OR cefamandole* OR cefapirin* OR cefatrizine* OR cefazaflur* OR cefazedone* OR cefazolin* OR cefbuperazone* OR cefcanel* OR cefcapene* OR cefclidin* OR cefdaloxime* OR cefdinir* OR cefditoren* OR cefepime* OR cefetamet* OR cefetecol* OR cefiderocol* OR cefixime* OR cefluprenam* OR cefmatilen* OR cefmenoxime* OR cefmetazole* OR cefminox* OR cefodizime* OR cefonicid* OR cefoperazone* OR ceforanide* OR cefoselis* OR cefotaxime* OR cefotetan* OR cefotiam* OR cefovecin* OR cefoxitin* OR cefozopran* OR cefpimizole* OR cefpiramide* OR cefpirome* OR cefpodoxime* OR cefprozil* OR cefquinome* OR cefradine* OR cefroxadine* OR cefsulodin* OR ceftaroline* OR ceftazidime* OR cefteram* OR ceftezole* OR ceftibuten* OR ceftiofur* OR ceftizoxime* OR ceftobiprole* OR ceftolozane* OR ceftriaxone* OR cefuroxime* OR cefuzonam* OR cephalosporin* OR cephamycin* OR cloxacillin* OR cyclacillin* OR deacetoxycephalosporin* OR deacetylcefotaxime* OR deacetylcephalosporin* OR dicloxacillin* OR enmetazobactam* OR epicillin* OR fleroxacin-deacetylcefotaxime-ester* OR flomoxef* OR flucloxacillin* OR flumoxil* OR fomidacillin* OR furbenicillin* OR fuzlocillin* OR gloximonam* OR hetacillin* OR isopenicillin* OR latamoxef* OR lenampicillin* OR loracarbef* OR mecillinam* OR metampicillin* OR meticillin* OR mezlocillin* OR miraxid* OR nafcillin* OR nitrocefin* OR optocillin* OR oxacillin* OR penamecillin* OR penethamate* OR penicillic-acid* OR penicillin* OR penicilloic-acid* OR pheneticillin* OR piperacillin* OR pivampicillin* OR pivmecillinam* OR propicillin* OR quinacillin* OR retacillin* OR sulbenicillin* OR sulfazecin* OR talampicillin* OR tameticillin* OR temocillin* OR thiophenoxycefalotin* OR ticarcillin* OR tigemonam* OR tobicillin* OR triplopen* OR ureidopenicillin*).ab,ti.) AND (Protein Binding / OR ((binding ADJ6 protein) OR Free-fraction* OR bound-fraction* OR unbound-fraction*).ab,ti.) AND (exp Body Fluids/ OR exp Hematologic Tests/ OR Urinalysis/ OR Protein Array Analysis/ OR Sequence Analysis, Protein/ OR (((body OR ascites* OR blister* OR cerebrospinal* OR dentinal* OR duodenum* OR edema* OR epithelial-lining* OR extracellular* OR extravascular* OR follicle* OR intestine* OR intracellular* OR lung-extravascular* OR lung* OR ovary-follicle* OR peritoneal* OR pleura* OR synovial* OR transcellular* OR uterus* OR wound* OR interstitial*) ADJ3 fluid*) OR blood* OR plasma OR serum* OR endolymph* OR perilymph* OR lymph* OR hemolymph* OR urine* OR aqueous-humor*).ab,ti.) NOT (exp *Drug Resistance, Microbial / OR (resistan*).ti.) NOT (exp animals/ NOT humans/) NOT (In Vitro Techniques / OR in-vitro.ti.)

**Web of science Core Collection n=740**

TS=(((beta-lactam OR beta-lactams OR β-lactam OR β-lactams OR biapenem* OR brobactam* OR carbapenem* OR clavulanate* OR clavulanic-acid* OR doripenem* OR durlobactam* OR ertapenem* OR fropenem* OR imipenem* OR l-786392* OR lenapenem* OR meropenem* OR monobactam* OR nacubactam* OR nocardicin-A* OR nocardicin-E* OR nocardicinic-acid* OR panipenem* OR penicillin* OR pirazmonam* OR razupenem* OR ritipenem* OR sanfetrinem* OR sulbactam* OR sulopenem* OR sultamicillin* OR taniborbactam* OR tazobactam* OR tebipenem* OR thienamycin* OR timentin* OR tomopenem* OR tribactam* OR trinem* OR aztreonam* OR adicillin* OR almecillin* OR aminopenicillin* OR amoxicillin* OR ampicillin* OR ancremonam* OR apalcillin* OR aspoxicillin* OR azidocillin* OR azlocillin* OR bacampicillin* OR bacmecillinam* OR carbacephem* OR carbenicillin* OR carfecillin* OR carindacillin* OR carumonam* OR cefacetrile* OR cefaclor* OR cefadroxil* OR cefalexin* OR cefaloglycin* OR cefaloram* OR cefaloridine* OR cefalotin* OR cefamandole* OR cefapirin* OR cefatrizine* OR cefazaflur* OR cefazedone* OR cefazolin* OR cefbuperazone* OR cefcanel* OR cefcapene* OR cefclidin* OR cefdaloxime* OR cefdinir* OR cefditoren* OR cefepime* OR cefetamet* OR cefetecol* OR cefiderocol* OR cefixime* OR cefluprenam* OR cefmatilen* OR cefmenoxime* OR cefmetazole* OR cefminox* OR cefodizime* OR cefonicid* OR cefoperazone* OR ceforanide* OR cefoselis* OR cefotaxime* OR cefotetan* OR cefotiam* OR cefovecin* OR cefoxitin* OR cefozopran* OR cefpimizole* OR cefpiramide* OR cefpirome* OR cefpodoxime* OR cefprozil* OR cefquinome* OR cefradine* OR cefroxadine* OR cefsulodin* OR ceftaroline* OR ceftazidime* OR cefteram* OR ceftezole* OR ceftibuten* OR ceftiofur* OR ceftizoxime* OR ceftobiprole* OR ceftolozane* OR ceftriaxone* OR cefuroxime* OR cefuzonam* OR cephalosporin* OR cephamycin* OR cloxacillin* OR cyclacillin* OR deacetoxycephalosporin* OR deacetylcefotaxime* OR deacetylcephalosporin* OR dicloxacillin* OR enmetazobactam* OR epicillin* OR fleroxacin-deacetylcefotaxime-ester* OR flomoxef* OR flucloxacillin* OR flumoxil* OR fomidacillin* OR furbenicillin* OR fuzlocillin* OR gloximonam* OR hetacillin* OR isopenicillin* OR latamoxef* OR lenampicillin* OR loracarbef* OR mecillinam* OR metampicillin* OR meticillin* OR mezlocillin* OR miraxid* OR nafcillin* OR nitrocefin* OR optocillin* OR oxacillin* OR penamecillin* OR penethamate* OR penicillic-acid* OR penicillin* OR penicilloic-acid* OR pheneticillin* OR piperacillin* OR pivampicillin* OR pivmecillinam* OR propicillin* OR quinacillin* OR retacillin* OR sulbenicillin* OR sulfazecin* OR talampicillin* OR tameticillin* OR temocillin* OR thiophenoxycefalotin* OR ticarcillin* OR tigemonam* OR tobicillin* OR triplopen* OR ureidopenicillin*)) AND (((binding NEAR/5 protein) OR Free-fraction* OR bound-fraction* OR unbound-fraction*)) AND ((((body OR ascites* OR blister* OR cerebrospinal* OR dentinal* OR duodenum* OR edema* OR epithelial-lining* OR extracellular* OR extravascular* OR follicle* OR intestine* OR intracellular* OR lung-extravascular* OR lung* OR ovary-follicle* OR peritoneal* OR pleura* OR synovial* OR transcellular* OR uterus* OR wound* OR interstitial*) NEAR/2 fluid*) OR blood* OR plasma OR serum* OR endolymph* OR perilymph* OR lymph* OR hemolymph* OR urine* OR aqueous-humor*))) NOT TI=(resistan* OR in-vitro) AND DT=(article)

**Cochrane CENTRAL register of Trials n=59**

((beta NEXT lactam OR beta NEXT lactams OR β NEXT lactam OR β NEXT lactams OR biapenem* OR brobactam* OR carbapenem* OR clavulanate* OR clavulanic NEXT acid* OR doripenem* OR durlobactam* OR ertapenem* OR fropenem* OR imipenem* OR l NEXT 786392* OR lenapenem* OR meropenem* OR monobactam* OR nacubactam* OR nocardicin NEXT A* OR nocardicin NEXT E* OR nocardicinic NEXT acid* OR panipenem* OR penicillin* OR pirazmonam* OR razupenem* OR ritipenem* OR sanfetrinem* OR sulbactam* OR sulopenem* OR sultamicillin* OR taniborbactam* OR tazobactam* OR tebipenem* OR thienamycin* OR timentin* OR tomopenem* OR tribactam* OR trinem* OR aztreonam* OR adicillin* OR almecillin* OR aminopenicillin* OR amoxicillin* OR ampicillin* OR ancremonam* OR apalcillin* OR aspoxicillin* OR azidocillin* OR azlocillin* OR bacampicillin* OR bacmecillinam* OR carbacephem* OR carbenicillin* OR carfecillin* OR carindacillin* OR carumonam* OR cefacetrile* OR cefaclor* OR cefadroxil* OR cefalexin* OR cefaloglycin* OR cefaloram* OR cefaloridine* OR cefalotin* OR cefamandole* OR cefapirin* OR cefatrizine* OR cefazaflur* OR cefazedone* OR cefazolin* OR cefbuperazone* OR cefcanel* OR cefcapene* OR cefclidin* OR cefdaloxime* OR cefdinir* OR cefditoren* OR cefepime* OR cefetamet* OR cefetecol* OR cefiderocol* OR cefixime* OR cefluprenam* OR cefmatilen* OR cefmenoxime* OR cefmetazole* OR cefminox* OR cefodizime* OR cefonicid* OR cefoperazone* OR ceforanide* OR cefoselis* OR cefotaxime* OR cefotetan* OR cefotiam* OR cefovecin* OR cefoxitin* OR cefozopran* OR cefpimizole* OR cefpiramide* OR cefpirome* OR cefpodoxime* OR cefprozil* OR cefquinome* OR cefradine* OR cefroxadine* OR cefsulodin* OR ceftaroline* OR ceftazidime* OR cefteram* OR ceftezole* OR ceftibuten* OR ceftiofur* OR ceftizoxime* OR ceftobiprole* OR ceftolozane* OR ceftriaxone* OR cefuroxime* OR cefuzonam* OR cephalosporin* OR cephamycin* OR cloxacillin* OR cyclacillin* OR deacetoxycephalosporin* OR deacetylcefotaxime* OR deacetylcephalosporin* OR dicloxacillin* OR enmetazobactam* OR epicillin* OR fleroxacin NEXT deacetylcefotaxime NEXT ester* OR flomoxef* OR flucloxacillin* OR flumoxil* OR fomidacillin* OR furbenicillin* OR fuzlocillin* OR gloximonam* OR hetacillin* OR isopenicillin* OR latamoxef* OR lenampicillin* OR loracarbef* OR mecillinam* OR metampicillin* OR meticillin* OR mezlocillin* OR miraxid* OR nafcillin* OR nitrocefin* OR optocillin* OR oxacillin* OR penamecillin* OR penethamate* OR penicillic NEXT acid* OR penicillin* OR penicilloic NEXT acid* OR pheneticillin* OR piperacillin* OR pivampicillin* OR pivmecillinam* OR propicillin* OR quinacillin* OR retacillin* OR sulbenicillin* OR sulfazecin* OR talampicillin* OR tameticillin* OR temocillin* OR thiophenoxycefalotin* OR ticarcillin* OR tigemonam* OR tobicillin* OR triplopen* OR ureidopenicillin*):ab,ti) AND (((binding NEAR/6 protein) OR Free NEXT fraction* OR bound NEXT fraction* OR unbound NEXT fraction*):ab,ti) AND ((((body OR ascites* OR blister* OR cerebrospinal* OR dentinal* OR duodenum* OR edema* OR epithelial NEXT lining* OR extracellular* OR extravascular* OR follicle* OR intestine* OR intracellular* OR lung NEXT extravascular* OR lung* OR ovary NEXT follicle* OR peritoneal* OR pleura* OR synovial* OR transcellular* OR uterus* OR wound* OR interstitial*) NEAR/3 fluid*) OR blood* OR plasma OR serum* OR endolymph* OR perilymph* OR lymph* OR hemolymph* OR urine* OR aqueous NEXT humor*):ab,ti) NOT ((resistan*):ti)
